# Multiscale analysis for patterns of Zika virus genotype emergence, spread, and consequence

**DOI:** 10.1371/journal.pone.0225699

**Published:** 2019-12-06

**Authors:** Monica K. Borucki, Nicole M. Collette, Lark L. Coffey, Koen K. A. Van Rompay, Mona H. Hwang, James B. Thissen, Jonathan E. Allen, Adam T. Zemla

**Affiliations:** 1 Physical Life Sciences Directorate, Lawrence Livermore National Laboratory, Livermore, California, United States of America; 2 Department of Pathology, Microbiology and Immunology, School of Veterinary Medicine, University of California Davis, Davis, California, United States of America; 3 California National Primate Research Center, University of California Davis, Davis, California, United States of America; 4 Computations Directorate, Lawrence Livermore National Laboratory, Livermore, California, United States of America; Centers for Disease Control and Prevention, UNITED STATES

## Abstract

The question of how Zika virus (ZIKV) changed from a seemingly mild virus to a human pathogen capable of microcephaly and sexual transmission remains unanswered. The unexpected emergence of ZIKV’s pathogenicity and capacity for sexual transmission may be due to genetic changes, and future changes in phenotype may continue to occur as the virus expands its geographic range. Alternatively, the sheer size of the 2015–16 epidemic may have brought attention to a pre-existing virulent ZIKV phenotype in a highly susceptible population. Thus, it is important to identify patterns of genetic change that may yield a better understanding of ZIKV emergence and evolution. However, because ZIKV has an RNA genome and a polymerase incapable of proofreading, it undergoes rapid mutation which makes it difficult to identify combinations of mutations associated with viral emergence. As next generation sequencing technology has allowed whole genome consensus and variant sequence data to be generated for numerous virus samples, the task of analyzing these genomes for patterns of mutation has become more complex. However, understanding which combinations of mutations spread widely and become established in new geographic regions versus those that disappear relatively quickly is essential for defining the trajectory of an ongoing epidemic. In this study, multiscale analysis of the wealth of genomic data generated over the course of the epidemic combined with *in vivo* laboratory data allowed trends in mutations and outbreak trajectory to be assessed. Mutations were detected throughout the genome via deep sequencing, and many variants appeared in multiple samples and in some cases become consensus. Similarly, amino acids that were previously consensus in pre-outbreak samples were detected as low frequency variants in epidemic strains. Protein structural models indicate that most of the mutations associated with the epidemic transmission occur on the exposed surface of viral proteins. At the macroscale level, consensus data was organized into large and interactive databases to allow the spread of individual mutations and combinations of mutations to be visualized and assessed for temporal and geographical patterns. Thus, the use of multiscale modeling for identifying mutations or combinations of mutations that impact epidemic transmission and phenotypic impact can aid the formation of hypotheses which can then be tested using reverse genetics.

## Introduction

First isolated in 1947 from a rhesus macaque in Uganda, Zika virus (ZIKV) was largely ignored until 2015 when a large outbreak of ZIKV infections in Brazil was linked to a dramatic increase in microcephaly cases [1–6]. ZIKV has a number of unexpected attributes for an arthropod-borne virus (“arbovirus”). In addition to the unusual propensity of ZIKV to cause microcephaly and other births defects, ZIKV can be transmitted sexually and has been shown to persist in the male and female reproductive systems [7–12].

ZIKV belongs to the family *Flaviviridae*, genus *Flavivirus*, which includes other mosquito-borne pathogens such as West Nile virus (WNV), dengue virus (DENV), yellow fever virus (YFV), and Japanese encephalitis virus (JEV) as well as tick-borne pathogens such as tick-borne encephalitis virus and Omsk hemorrhagic fever virus [13]. Similar to DENV and YFV, the ZIKV lifecycle includes *Aedes* mosquitoes and primates [1,14–17], whereas WNV is transmitted by a wider range of mosquito species, primarily in the genus *Culex* [18], and birds serve as reservoirs [19].

ZIKV has a 10.7 kb genome of single-stranded RNA encoding ten proteins. The genome is translated into a polyprotein of about 3300 amino acids which is cleaved by viral and host proteases into three structural proteins (C, Pr/M, and E) and seven nonstructural (NS1, NS2A, NS2B, NS3, NS4A, NS4B, and NS5) proteins. The structural proteins form the viral particle whereas the nonstructural proteins form a complex responsible for viral replication. Both structural and nonstructural proteins interact with the host immune response [20–24].

As is the case with all viruses with RNA genomes, ZIKV mutates rapidly due to its highly error prone RNA-dependent-RNA-polymerase. The high mutation rate of RNA viruses increases the ability of these viruses to adapt to diverse hosts and cause new human and zoonotic diseases [25]. Among RNA viruses, arboviruses such as ZIKV are notable for having a relatively conserved consensus sequence, which changes more slowly compared to other RNA viruses due to the requirement for arboviruses to replicate efficiently in both vertebrate and invertebrate hosts [26]. However, the relatively limited evolution rate of the consensus sequence masks the presence of viral variants within the sample that influence viral virulence and transmission cycles and which are constantly in flux in response to environmental conditions [27,28]. In fact, viral virulence may change in the absence of mutation in the consensus sequence but rather due to changes in the composition of the intra-host subconsensus genotypes (referred to as “mutant spectra” or “quasispecies”). Thus, deep sequencing of the genetic variant genotypes is necessary to understand the mechanisms used by the virus to change phenotype, including virulence, tissue tropism, or host range. Importantly, identifying the variant genotypes circulating within infected hosts allows early detection of genotypes that may evade countermeasures and diagnostic assays and impact viral phenotype.

In this study multiscale analysis of the wealth of genomic data generated over the course of the epidemic and available in public databases was combined with *in vivo* laboratory data. The objective of this study was to examine viral emergence at multiple scales: the microscale (molecular) and macroscale (temporal and special dispersion of mutations) to enable trends in mutations and outbreak trajectory to be assessed. Using the recent ZIKV epidemic as a model, we mined both consensus and subconsensus sequence data derived from *in vivo* infections and from Illumina read data available in GenBank in an effort to define patterns of mutations that may impact viral emergence and virulence. The genomic data were queried for the presence of consensus and subconsensus variants that persist over time and increase in prevalence. Because whole genomic sequence data was available for over 400 genomes associated with the outbreak, a process was developed for organizing large sets of sequence data, thus enabling patterns of emergence and spread of mutations associated with the 2015–2016 outbreak to be readily visualized. Comparative analyses of constructed protein structure models of different ZIKV variants were used to identify mutations likely to modify protein structure conformation. This approach provides insight into which mutations or combinations of mutations should be further investigated using detailed structural modeling and reverse genetics in functional assays.

## Results and discussion

ZIKV sequence data from publicly available databases and generated as part of this study were organized to look for patterns of mutation at multiscale levels. At the macroscale level, consensus sequence data were organized into large and interactive databases to allow the spread of individual mutations and combinations of mutations to be visualized and assessed for temporal and geographical patterns. At the microscale level, data from deep Illumina sequencing was used to analyze subconsensus mutations (“variants”) for the presence of epidemic-associated mutations in pre-epidemic samples (2006–2014), and for persistent and prevalent variants that may play a role in quasispecies phenotype and/or the emergence of novel genotypes. Finally, mutations were examined at the protein structure level to look for molecular characteristics of mutations associated with epidemic-associated mutations.

### Trends of mutation prevalence and persistence

Analysis of ZIKV genomes show distinct patterns of emergence of nonsynonymous mutations in the years prior to the epidemic, as has been previously described [6,29,30], and superbly documented in the nextstrain.org Zika website (https://nextstrain.org/zika) [31]. However, as the number of genome sequences in databases such as GenBank increase well into the hundreds, patterns of dispersal become harder to decipher using conventional methods such as dendrograms, and it is difficult to discern the impact of gaps in sequence data. Thus, a process was developed for organizing large sets of sequence data enabling patterns of emergence and spread of mutations associated with the 2015–2016 outbreak to be readily visualized, as well as gaps in whole genome sequence data to be shown.

Hundreds of mutations that emerged in the genome of ZIKV as the Asian lineage spread from French Polynesia to Haiti and Brazil, and then to other countries in South, Central, and North America, and beyond [6,29,32–34], were first assembled into a database using all ZIKV whole genome sequence data deposited in GenBank and ViPR (Virus Pathogen Resource; viprbrc.org) databases prior to July 2018. A set of 408 complete genome sequences was collected and evaluated, and 544 nonsynonymous mutations were identified for further analysis. Mutations in the 5’ and 3’ UTR of the genome were not included in the database, nor were synonymous mutations. S1 Table shows nonsynonymous mutations that were observed in at least three sequences and that were both prevalent and persistent as the outbreak expanded. S2 Table provides the genome coordinates as nucleotide site (according to reference sequence PRVABC-59, accession # KU501215), polyprotein residue, protein residue information, and data from protein structural analysis to allow data from different publications to be readily compared. In Tables 1–4, subsets of genome variants were organized according to geographical location and number of whole genome sequences in GenBank or ViPR that carry the variant, illustrating trends of co-dispersal of mutations associated with the 2015–2016 epidemic. Because it is not known if Asian lineage strains circulating prior to the French Polynesia outbreak are capable of causing microcephaly in humans, genomes from Thailand 2006, Cambodia 2010, Philippines 2012, and Thailand 2013 are included in the analysis as “pre-emergent strains” (in the context of the 2015–16 epidemic) and compared along with the later Asian genomes associated with epidemic. Asian strain Malaysia 1966 was used as a distant progenitor for comparison and considered to be unassociated with the recent emergence of ZIKV.

**Table 1:** Dispersal patterns of select mutations according to country and year: Comparison of patterns of emergence of select persistent mutations in the Pr and Env genes. The number of whole genome sequences present in GenBank and ViPR databases for pre-emergent and epidemic-associated mutations were tabulated to allow easy visualization of similarities in time, location, and number of cases between mutations. Mutations identified as increasing in prevalence and/or persistence can be further visualized for trajectory. The first column identifies each mutation according to nucleotide (nt) site: codon: protein: residue #: amino acid, followed by the year and total number of samples sequenced that year (N/Year). Cells are colored according to number (red, yellow, green: high, medium, low). S1 Table lists all nonsynonymous mutations detected in 3 or more sequences. Country names are abbreviated using the first 5 letters of the name or common abbreviation. AA—amino acid; Impor—imported case.

**Nt**	**Codon**	**Gene**	**Residue**	**AA**	**Year**	**N/Year**	**Thail**	**Cambo**	**Indon**	**Phili**	**FrPol**	**Haiti**	**Brazi**	**FrGui**	**Surin**	**Colom**	**Panam**	**Hondu**	**Guate**	**Mexic**	**PuRic**
473	GCG	Pr	1	A	2006	0	-	-	-	-	-	-	-	-	-	-	-	-	-	-	-
					2010	0	-	-	-	-	-	-	-	-	-	-	-	-	-	-	-
					2012	0	-	-	-	-	-	-	-	-	-	-	-	-	-	-	-
					2013	12	3	-	-	-	9	-	-	-	-	-	-	-	-	-	-
					2014	13	1	-	1	-	4	7	-	-	-	-	-	-	-	-	-
					2015	67	1	-	-	-	-	-	20	1	1	30	3	1	2	3	4
					2016	295	4	-	-	-	-	3	54	-	2	20	1	22	3	51	2
					2017	10	1	-	-	-	-	-	-	-	-	-	-	-	-	1	-
																					
521	AAC	Pr	17	N	2006	0	-	-	-	-	-	-	-	-	-	-	-	-	-	-	-
					2010	0	-	-	-	-	-	-	-	-	-	-	-	-	-	-	-
					2012	0	-	-	-	-	-	-	-	-	-	-	-	-	-	-	-
					2013	9	-	-	-	-	9	-	-	-	-	-	-	-	-	-	-
					2014	11	-	-	-	-	4	7	-	-	-	-	-	-	-	-	-
					2015	66	-	-	-	-	-	-	20	1	1	30	3	1	2	3	4
					2016	284	-	-	-	-	-	3	51	-	2	18	1	22	3	51	2
					2017	9	-	-	-	-	-	-	-	-	-	-	-	-	-	1	-
																					
2153	GAG	Env	393	E	2006	1	1	-	-	-	-	-	-	-	-	-	-	-	-	-	-
					2010	0	-	-	-	-	-	-	-	-	-	-	-	-	-	-	-
					2012	1	-	-	-	1	-	-	-	-	-	-	-	-	-	-	-
					2013	12	3	-	-	-	9	-	-	-	-	-	-	-	-	-	-
					2014	13	1	-	1	-	4	7	-	-	-	-	-	-	-	-	-
					2015	67	1	-	-	-	-	-	20	1	1	30	3	1	2	3	4
					2016	294	4	-	-	2	-	3	53	-	2	20	1	21	3	51	2
					2017	10	1	-	-	-	-	-	-	-	-	-	-	-	-	1	-
																					
2393	ATG	Env	473	M	2006	0	-	-	-	-	-	-	-	-	-	-	-	-	-	-	-
					2010	0	-	-	-	-	-	-	-	-	-	-	-	-	-	-	-
					2012	0	-	-	-	-	-	-	-	-	-	-	-	-	-	-	-
					2013	12	3	-	-	-	9	-	-	-	-	-	-	-	-	-	-
					2014	13	1	-	1	-	4	7	-	-	-	-	-	-	-	-	-
					2015	67	1	-	-	-	-	-	20	1	1	30	3	1	2	3	4
					2016	286	3	-	-	-	-	3	49	-	2	19	1	22	3	50	2
					2017	9	1	-	-	-	-	-	-	-	-	-	-	-	-	1	-
																					
2435	ATG	Env	487	M	2006	1	1	-	-	-	-	-	-	-	-	-	-	-	-	-	-
					2010	1	-	1	-	-	-	-	-	-	-	-	-	-	-	-	-
					2012	0	-	-	-	-	-	-	-	-	-	-	-	-	-	-	-
					2013	12	3	-	-	-	9	-	-	-	-	-	-	-	-	-	-
					2014	13	1	-	1	-	4	7	-	-	-	-	-	-	-	-	-
					2015	67	1	-	-	-	-	-	20	1	1	30	3	1	2	3	4
					2016	286	3	-	-	-	-	3	49	-	2	19	1	22	3	50	2
					2017	9	1	-	-	-	-	-	-	-	-	-	-	-	-	1	-
**Nt**	**Codon**	**Gene**	**Residue**	**AA**	**Year**	**N/Year**	**Venez**	**Ecuad**	**Peru**	**Nicar**	**Guade**	**DoRep**	**USA**	**Jamai**	**Marti**	**Cuba**	**Fiji**	**Samoa**	**Tonga**	**Impor**	
473	GCG	Pr	1	A	2006	0	-	-	-	-	-	-	-	-	-	-	-	-	-	-	
					2010	0	-	-	-	-	-	-	-	-	-	-	-	-	-	-	
					2012	0	-	-	-	-	-	-	-	-	-	-	-	-	-	-	
					2013	12	-	-	-	-	-	-	-	-	-	-	-	-	-	-	
					2014	13	-	-	-	-	-	-	-	-	-	-	-	-	-	-	
					2015	67	-	-	-	-	-	-	-	-	1	-	-	-	-	-	
					2016	295	10	3	2	20	1	29	51	4	1	-	4	4	1	3	
					2017	10	-	-	-	-	-	-	-	-	-	8	-	-	-	-	
																					
521	AAC	Pr	17	N	2006	0	-	-	-	-	-	-	-	-	-	-	-	-	-	-	
					2010	0	-	-	-	-	-	-	-	-	-	-	-	-	-	-	
					2012	0	-	-	-	-	-	-	-	-	-	-	-	-	-	-	
					2013	9	-	-	-	-	-	-	-	-	-	-	-	-	-	-	
					2014	11	-	-	-	-	-	-	-	-	-	-	-	-	-	-	
					2015	66	-	-	-	-	-	-	-	-	1	-	-	-	-	-	
					2016	284	10	3	2	20	1	29	50	3	1	-	4	4	1	3	
					2017	9	-	-	-	-	-	-	-	-	-	8	-	-	-	-	
																					
2153	GAG	Env	393	E	2006	1	-	-	-	-	-	-	-	-	-	-	-	-	-	-	
					2010	0	-	-	-	-	-	-	-	-	-	-	-	-	-	-	
					2012	1	-	-	-	-	-	-	-	-	-	-	-	-	-	-	
					2013	12	-	-	-	-	-	-	-	-	-	-	-	-	-	-	
					2014	13	-	-	-	-	-	-	-	-	-	-	-	-	-	-	
					2015	67	-	-	-	-	-	-	-	-	1	-	-	-	-	-	
					2016	294	10	3	2	20	1	28	52	4	-	-	4	4	1	3	
					2017	10	-	-	-	-	-	-	-	-	-	8	-	-	-	-	
																					
2393	ATG	Env	473	M	2006	0	-	-	-	-	-	-	-	-	-	-	-	-	-	-	
					2010	0	-	-	-	-	-	-	-	-	-	-	-	-	-	-	
					2012	0	-	-	-	-	-	-	-	-	-	-	-	-	-	-	
					2013	12	-	-	-	-	-	-	-	-	-	-	-	-	-	-	
					2014	13	-	-	-	-	-	-	-	-	-	-	-	-	-	-	
					2015	67	-	-	-	-	-	-	-	-	1	-	-	-	-	-	
					2016	286	10	3	2	19	1	28	54	2	1	-	4	4	1	3	
					2017	9	-	-	-	-	-	-	-	-	-	7	-	-	-	-	
																					
2435	ATG	Env	487	M	2006	1	-	-	-	-	-	-	-	-	-	-	-	-	-	-	
					2010	1	-	-	-	-	-	-	-	-	-	-	-	-	-	-	
					2012	0	-	-	-	-	-	-	-	-	-	-	-	-	-	-	
					2013	12	-	-	-	-	-	-	-	-	-	-	-	-	-	-	
					2014	13	-	-	-	-	-	-	-	-	-	-	-	-	-	-	
					2015	67	-	-	-	-	-	-	-	-	1	-	-	-	-	-	
					2016	286	10	3	2	19	1	28	54	2	1	-	4	4	1	3	
					2017	9	-	-	-	-	-	-	-	-	-	7	-	-	-	-	

**Table 2:** Dispersal patterns of select mutations according to country and year: Comparison of patterns of emergence of select persistent mutations in the NS1 gene.

**Nt**	**Codon**	**Gene**	**Residue**	**AA**	**Year**	**N/Year**	**Thail**	**Cambo**	**Indon**	**Phili**	**FrPol**	**Haiti**	**Brazi**	**FrGui**	**Surin**	**Colom**	**Panam**	**Hondu**	**Guate**	**Mexic**	**PuRic**
2786	GCT	NS1	100	A	2006	0	-	-	-	-	-	-	-	-	-	-	-	-	-	-	-
					2010	0	-	-	-	-	-	-	-	-	-	-	-	-	-	-	-
					2012	0	-	-	-	-	-	-	-	-	-	-	-	-	-	-	-
					2013	0	-	-	-	-	-	-	-	-	-	-	-	-	-	-	-
					2014	0	-	-	-	-	-	-	-	-	-	-	-	-	-	-	-
					2015	6	-	-	-	-	-	-	-	-	-	-	-	1	2	3	-
					2016	88	-	-	-	-	-	-	-	-	-	-	-	20	3	43	-
					2017	5	-	-	-	-	-	-	-	-	-	-	-	-	-	1	-
																					
3050	GTC	NS1	188	V	2006	0	-	-	-	-	-	-	-	-	-	-	-	-	-	-	-
					2010	0	-	-	-	-	-	-	-	-	-	-	-	-	-	-	-
					2012	0	-	-	-	-	-	-	-	-	-	-	-	-	-	-	-
					2013	0	-	-	-	-	-	-	-	-	-	-	-	-	-	-	-
					2014	1	-	-	1	-	-	-	-	-	-	-	-	-	-	-	-
					2015	0	-	-	-	-	-	-	-	-	-	-	-	-	-	-	-
					2016	5	-	-	-	2	-	-	-	-	-	-	-	-	-	2	-
					2017	1	-	-	-	-	-	-	-	-	-	-	-	-	-	1	-
																					
3050	GTT	NS1	188	V	2006	0	-	-	-	-	-	-	-	-	-	-	-	-	-	-	-
					2010	0	-	-	-	-	-	-	-	-	-	-	-	-	-	-	-
					2012	0	-	-	-	-	-	-	-	-	-	-	-	-	-	-	-
					2013	12	3	-	-	-	9	-	-	-	-	-	-	-	-	-	-
					2014	12	1	-	-	-	4	7	-	-	-	-	-	-	-	-	-
					2015	67	1	-	-	-	-	-	20	1	1	30	3	1	2	3	4
					2016	285	4	-	-	-	-	3	53	-	2	19	1	20	3	47	2
					2017	9	1	-	-	-	-	-	-	-	-	-	-	-	-	-	-
																					
3278	ATG	NS1	264	M	2006	1	1	-	-	-	-	-	-	-	-	-	-	-	-	-	-
					2010	1	-	1	-	-	-	-	-	-	-	-	-	-	-	-	-
					2012	1	-	-	-	1	-	-	-	-	-	-	-	-	-	-	-
					2013	12	3	-	-	-	9	-	-	-	-	-	-	-	-	-	-
					2014	13	1	-	1	-	4	7	-	-	-	-	-	-	-	-	-
					2015	67	1	-	-	-	-	-	20	1	1	30	3	1	2	3	4
					2016	288	4	-	-	2	-	3	52	-	2	19	1	20	3	48	1
					2017	10	1	-	-	-	-	-	-	-	-	-	-	-	-	1	-
																					
3458	CAG	NS1	324	Q	2006	0	-	-	-	-	-	-	-	-	-	-	-	-	-	-	-
					2010	0	-	-	-	-	-	-	-	-	-	-	-	-	-	-	-
					2012	0	-	-	-	-	-	-	-	-	-	-	-	-	-	-	-
					2013	0	-	-	-	-	-	-	-	-	-	-	-	-	-	-	-
					2014	0	-	-	-	-	-	-	-	-	-	-	-	-	-	-	-
					2015	0	-	-	-	-	-	-	-	-	-	-	-	-	-	-	-
					2016	9	-	-	-	-	-	-	-	-	-	-	-	-	-	-	-
					2017	0	-	-	-	-	-	-	-	-	-	-	-	-	-	-	-
																					
3458	TGG	NS1	324	W	2006	0	-	-	-	-	-	-	-	-	-	-	-	-	-	-	-
					2010	0	-	-	-	-	-	-	-	-	-	-	-	-	-	-	-
					2012	0	-	-	-	-	-	-	-	-	-	-	-	-	-	-	-
					2013	0	-	-	-	-	-	-	-	-	-	-	-	-	-	-	-
					2014	0	-	-	-	-	-	-	-	-	-	-	-	-	-	-	-
					2015	35	-	-	-	-	-	-	1	-	-	30	3	-	-	-	-
					2016	27	-	-	-	-	-	-	-	-	-	17	1	-	-	2	-
					2017	0	-	-	-	-	-	-	-	-	-	-	-	-	-	-	-
																					
3533	GTG	NS1	349	V	2006	0	-	-	-	-	-	-	-	-	-	-	-	-	-	-	-
					2010	0	-	-	-	-	-	-	-	-	-	-	-	-	-	-	-
					2012	0	-	-	-	-	-	-	-	-	-	-	-	-	-	-	-
					2013	0	-	-	-	-	-	-	-	-	-	-	-	-	-	-	-
					2014	6	-	-	-	-	-	6	-	-	-	-	-	-	-	-	-
					2015	5	-	-	-	-	-	-	5	-	-	-	-	-	-	-	-
					2016	85	-	-	-	-	-	2	5	-	-	-	-	-	-	-	-
					2017	4	-	-	-	-	-	-	-	-	-	-	-	-	-	-	-
**Nt**	**Codon**	**Gene**	**Residue**	**AA**	**Year**	**N/Year**	**Venez**	**Ecuad**	**Peru**	**Nicar**	**Guade**	**DoRep**	**USA**	**Jamai**	**Marti**	**Cuba**	**Fiji**	**Samoa**	**Tonga**	**Impor**	
2786	GCT	NS1	100	A	2006	0	-	-	-	-	-	-	-	-	-	-	-	-	-	-	
					2010	0	-	-	-	-	-	-	-	-	-	-	-	-	-	-	
					2012	0	-	-	-	-	-	-	-	-	-	-	-	-	-	-	
					2013	0	-	-	-	-	-	-	-	-	-	-	-	-	-	-	
					2014	0	-	-	-	-	-	-	-	-	-	-	-	-	-	-	
					2015	6	-	-	-	-	-	-	-	-	-	-	-	-	-	-	
					2016	88	-	-	-	19	-	-	3	-	-	-	-	-	-	-	
					2017	5	-	-	-	-	-	-	-	-	-	4	-	-	-	-	
																					
3050	GTC	NS1	188	V	2006	0	-	-	-	-	-	-	-	-	-	-	-	-	-	-	
					2010	0	-	-	-	-	-	-	-	-	-	-	-	-	-	-	
					2012	0	-	-	-	-	-	-	-	-	-	-	-	-	-	-	
					2013	0	-	-	-	-	-	-	-	-	-	-	-	-	-	-	
					2014	1	-	-	-	-	-	-	-	-	-	-	-	-	-	-	
					2015	0	-	-	-	-	-	-	-	-	-	-	-	-	-	-	
					2016	5	-	-	-	-	-	-	1	-	-	-	-	-	-	-	
					2017	1	-	-	-	-	-	-	-	-	-	-	-	-	-	-	
																					
3050	GTT	NS1	188	V	2006	0	-	-	-	-	-	-	-	-	-	-	-	-	-	-	
					2010	0	-	-	-	-	-	-	-	-	-	-	-	-	-	-	
					2012	0	-	-	-	-	-	-	-	-	-	-	-	-	-	-	
					2013	12	-	-	-	-	-	-	-	-	-	-	-	-	-	-	
					2014	12	-	-	-	-	-	-	-	-	-	-	-	-	-	-	
					2015	67	-	-	-	-	-	-	-	-	1	-	-	-	-	-	
					2016	285	10	3	2	19	1	30	49	4	1	-	4	4	1	3	
					2017	9	-	-	-	-	-	-	-	-	-	8	-	-	-	-	
																					
3278	ATG	NS1	264	M	2006	1	-	-	-	-	-	-	-	-	-	-	-	-	-	-	
					2010	1	-	-	-	-	-	-	-	-	-	-	-	-	-	-	
					2012	1	-	-	-	-	-	-	-	-	-	-	-	-	-	-	
					2013	12	-	-	-	-	-	-	-	-	-	-	-	-	-	-	
					2014	13	-	-	-	-	-	-	-	-	-	-	-	-	-	-	
					2015	67	-	-	-	-	-	-	-	-	1	-	-	-	-	-	
					2016	288	10	3	2	18	1	29	53	4	1	-	4	4	1	3	
					2017	10	-	-	-	-	-	-	-	-	-	8	-	-	-	-	
																					
3458	CAG	NS1	324	Q	2006	0	-	-	-	-	-	-	-	-	-	-	-	-	-	-	
					2010	0	-	-	-	-	-	-	-	-	-	-	-	-	-	-	
					2012	0	-	-	-	-	-	-	-	-	-	-	-	-	-	-	
					2013	0	-	-	-	-	-	-	-	-	-	-	-	-	-	-	
					2014	0	-	-	-	-	-	-	-	-	-	-	-	-	-	-	
					2015	0	-	-	-	-	-	-	-	-	-	-	-	-	-	-	
					2016	9	1	-	-	-	-	-	-	-	-	-	3	4	-	1	
					2017	0	-	-	-	-	-	-	-	-	-	-	-	-	-	-	
																					
3458	TGG	NS1	324	W	2006	0	-	-	-	-	-	-	-	-	-	-	-	-	-	-	
					2010	0	-	-	-	-	-	-	-	-	-	-	-	-	-	-	
					2012	0	-	-	-	-	-	-	-	-	-	-	-	-	-	-	
					2013	0	-	-	-	-	-	-	-	-	-	-	-	-	-	-	
					2014	0	-	-	-	-	-	-	-	-	-	-	-	-	-	-	
					2015	35	-	-	-	-	-	-	-	-	1	-	-	-	-	-	
					2016	27	2	-	2	-	-	1	1	-	1	-	-	-	-	-	
					2017	0	-	-	-	-	-	-	-	-	-	-	-	-	-	-	
																					
3533	GTG	NS1	349	V	2006	0	-	-	-	-	-	-	-	-	-	-	-	-	-	-	
					2010	0	-	-	-	-	-	-	-	-	-	-	-	-	-	-	
					2012	0	-	-	-	-	-	-	-	-	-	-	-	-	-	-	
					2013	0	-	-	-	-	-	-	-	-	-	-	-	-	-	-	
					2014	6	-	-	-	-	-	-	-	-	-	-	-	-	-	-	
					2015	5	-	-	-	-	-	-	-	-	-	-	-	-	-	-	
					2016	85	-	3	-	-	1	27	45	2	-	-	-	-	-	-	
					2017	4	-	-	-	-	-	-	-	-	-	4	-	-	-	-	

**Table 3:** Dispersal patterns of select mutations according to country and year: Comparison of patterns of emergence of select persistent mutations in the NS3 gene.

**Nt**	**Codon**	**Gene**	**Residue**	**AA**	**Year**	**N/Year**	**Thail**	**Cambo**	**Indon**	**Phili**	**FrPol**	**Haiti**	**Brazi**	**FrGui**	**Surin**	**Colom**	**Panam**	**Hondu**	**Guate**	**Mexic**	**PuRic**
5810	CAT	NS3	400	H	2006	1	1	-	-	-	-	-	-	-	-	-	-	-	-	-	-
					2010	1	-	1	-	-	-	-	-	-	-	-	-	-	-	-	-
					2012	0	-	-	-	-	-	-	-	-	-	-	-	-	-	-	-
					2013	11	2	-	-	-	9	-	-	-	-	-	-	-	-	-	-
					2014	13	1	-	1	-	4	7	-	-	-	-	-	-	-	-	-
					2015	67	1	-	-	-	-	-	20	1	1	30	3	1	2	3	4
					2016	290	4	-	-	-	-	3	53	-	2	20	1	20	3	50	2
					2017	10	1	-	-	-	-	-	-	-	-	-	-	-	-	1	-
																					
6026	CTA	NS3	472	L	2006	0	-	-	-	-	-	-	-	-	-	-	-	-	-	-	-
					2010	0	-	-	-	-	-	-	-	-	-	-	-	-	-	-	-
					2012	0	-	-	-	-	-	-	-	-	-	-	-	-	-	-	-
					2013	0	-	-	-	-	-	-	-	-	-	-	-	-	-	-	-
					2014	0	-	-	-	-	-	-	-	-	-	-	-	-	-	-	-
					2015	0	-	-	-	-	-	-	-	-	-	-	-	-	-	-	-
					2016	11	-	-	-	-	-	-	-	-	-	-	-	6	-	-	-
					2017	0	-	-	-	-	-	-	-	-	-	-	-	-	-	-	-
																					
6026	CTG	NS3	472	L	2006	1	1	-	-	-	-	-	-	-	-	-	-	-	-	-	-
					2010	1	-	1	-	-	-	-	-	-	-	-	-	-	-	-	-
					2012	1	-	-	-	1	-	-	-	-	-	-	-	-	-	-	-
					2013	12	3	-	-	-	9	-	-	-	-	-	-	-	-	-	-
					2014	13	1	-	1	-	4	7	-	-	-	-	-	-	-	-	-
					2015	67	1	-	-	-	-	-	20	1	1	30	3	1	2	3	4
					2016	278	4	-	-	2	-	3	52	-	2	20	1	14	3	49	2
					2017	10	1	-	-	-	-	-	-	-	-	-	-	-	-	1	-
																					
6326	CTG	NS3	572	L	2006	0	-	-	-	-	-	-	-	-	-	-	-	-	-	-	-
					2010	0	-	-	-	-	-	-	-	-	-	-	-	-	-	-	-
					2012	0	-	-	-	-	-	-	-	-	-	-	-	-	-	-	-
					2013	0	-	-	-	-	-	-	-	-	-	-	-	-	-	-	-
					2014	0	-	-	-	-	-	-	-	-	-	-	-	-	-	-	-
					2015	6	-	-	-	-	-	-	-	-	-	-	-	1	2	3	-
					2016	94	-	-	-	-	-	-	-	-	-	-	-	20	3	49	-
					2017	4	-	-	-	-	-	-	-	-	-	-	-	-	-	1	-
																					
6362	CAC	NS3	584	H	2006	0	-	-	-	-	-	-	-	-	-	-	-	-	-	-	-
					2010	0	-	-	-	-	-	-	-	-	-	-	-	-	-	-	-
					2012	0	-	-	-	-	-	-	-	-	-	-	-	-	-	-	-
					2013	9	-	-	-	-	9	-	-	-	-	-	-	-	-	-	-
					2014	12	1	-	-	-	4	7	-	-	-	-	-	-	-	-	-
					2015	66	1	-	-	-	-	-	19	1	1	30	3	1	2	3	4
					2016	280	1	-	-	-	-	3	52	-	2	19	1	20	3	50	2
					2017	9	-	-	-	-	-	-	-	-	-	-	-	-	-	1	-
**Nt**	**Codon**	**Gene**	**Residue**	**AA**	**Year**	**N/Year**	**Venez**	**Ecuad**	**Peru**	**Nicar**	**Guade**	**DoRep**	**USA**	**Jamai**	**Marti**	**Cuba**	**Fiji**	**Samoa**	**Tonga**	**Impor**	
5810	CAT	NS3	400	H	2006	1	-	-	-	-	-	-	-	-	-	-	-	-	-	-	
					2010	1	-	-	-	-	-	-	-	-	-	-	-	-	-	-	
					2012	0	-	-	-	-	-	-	-	-	-	-	-	-	-	-	
					2013	11	-	-	-	-	-	-	-	-	-	-	-	-	-	-	
					2014	13	-	-	-	-	-	-	-	-	-	-	-	-	-	-	
					2015	67	-	-	-	-	-	-	-	-	1	-	-	-	-	-	
					2016	290	10	3	2	20	1	30	50	3	1	-	4	4	1	3	
					2017	10	-	-	-	-	-	-	-	-	-	8	-	-	-	-	
																					
6026	CTA	NS3	472	L	2006	0	-	-	-	-	-	-	-	-	-	-	-	-	-	-	
					2010	0	-	-	-	-	-	-	-	-	-	-	-	-	-	-	
					2012	0	-	-	-	-	-	-	-	-	-	-	-	-	-	-	
					2013	0	-	-	-	-	-	-	-	-	-	-	-	-	-	-	
					2014	0	-	-	-	-	-	-	-	-	-	-	-	-	-	-	
					2015	0	-	-	-	-	-	-	-	-	-	-	-	-	-	-	
					2016	11	-	-	-	5	-	-	-	-	-	-	-	-	-	-	
					2017	0	-	-	-	-	-	-	-	-	-	-	-	-	-	-	
																					
6026	CTG	NS3	472	L	2006	1	-	-	-	-	-	-	-	-	-	-	-	-	-	-	
					2010	1	-	-	-	-	-	-	-	-	-	-	-	-	-	-	
					2012	1	-	-	-	-	-	-	-	-	-	-	-	-	-	-	
					2013	12	-	-	-	-	-	-	-	-	-	-	-	-	-	-	
					2014	13	-	-	-	-	-	-	-	-	-	-	-	-	-	-	
					2015	67	-	-	-	-	-	-	-	-	1	-	-	-	-	-	
					2016	278	10	3	2	14	1	28	52	3	1	-	4	4	1	3	
					2017	10	-	-	-	-	-	-	-	-	-	8	-	-	-	-	
																					
6326	CTG	NS3	572	L	2006	0	-	-	-	-	-	-	-	-	-	-	-	-	-	-	
					2010	0	-	-	-	-	-	-	-	-	-	-	-	-	-	-	
					2012	0	-	-	-	-	-	-	-	-	-	-	-	-	-	-	
					2013	0	-	-	-	-	-	-	-	-	-	-	-	-	-	-	
					2014	0	-	-	-	-	-	-	-	-	-	-	-	-	-	-	
					2015	6	-	-	-	-	-	-	-	-	-	-	-	-	-	-	
					2016	94	-	-	-	19	-	-	3	-	-	-	-	-	-	-	
					2017	4	-	-	-	-	-	-	-	-	-	3	-	-	-	-	
																					
6362	CAC	NS3	584	H	2006	0	-	-	-	-	-	-	-	-	-	-	-	-	-	-	
					2010	0	-	-	-	-	-	-	-	-	-	-	-	-	-	-	
					2012	0	-	-	-	-	-	-	-	-	-	-	-	-	-	-	
					2013	9	-	-	-	-	-	-	-	-	-	-	-	-	-	-	
					2014	12	-	-	-	-	-	-	-	-	-	-	-	-	-	-	
					2015	66	-	-	-	-	-	-	-	-	1	-	-	-	-	-	
					2016	280	10	3	2	17	1	28	50	3	1	-	4	4	1	3	
					2017	9	-	-	-	-	-	-	-	-	-	8	-	-	-	-	

**Table 4:** Dispersal patterns of select mutations according to country and year: Comparison of patterns of emergence of select persistent mutations in the NS5 gene.

**Nt**	**Codon**	**Gene**	**Residue**	**AA**	**Year**	**N/Year**	**Thail**	**Cambo**	**Indon**	**Phili**	**FrPol**	**Haiti**	**Brazi**	**FrGui**	**Surin**	**Colom**	**Panam**	**Hondu**	**Guate**	**Mexic**	**PuRic**
8006	ATG	NS5	114	M	2006	1	1	-	-	-	-	-	-	-	-	-	-	-	-	-	-
					2010	1	-	1	-	-	-	-	-	-	-	-	-	-	-	-	-
					2012	1	-	-	-	1	-	-	-	-	-	-	-	-	-	-	-
					2013	12	3	-	-	-	9	-	-	-	-	-	-	-	-	-	-
					2014	6	1	-	1	-	4	-	-	-	-	-	-	-	-	-	-
					2015	1	1	-	-	-	-	-	-	-	-	-	-	-	-	-	-
					2016	17	4	-	-	2	-	-	1	-	-	-	-	-	-	-	-
					2017	1	1	-	-	-	-	-	-	-	#NULL!	-	-	-	-	-	-
																					
8006	GTG	NS5	114	V	2006	0	-	-	-	-	-	-	-	-	-	-	-	-	-	-	-
					2010	0	-	-	-	-	-	-	-	-	-	-	-	-	-	-	-
					2012	0	-	-	-	-	-	-	-	-	-	-	-	-	-	-	-
					2013	0	-	-	-	-	-	-	-	-	-	-	-	-	-	-	-
					2014	7	-	-	-	-	-	7	-	-	-	-	-	-	-	-	-
					2015	65	-	-	-	-	-	-	19	1	1	30	3	1	2	3	4
					2016	256	-	-	-	-	-	3	44	-	2	20	1	19	3	49	2
					2017	7	-	-	-	-	-	-	-	-	-	-	-	-	-	1	-
																					
8081	CCG	NS5	139	P	2006	1	1	-	-	-	-	-	-	-	-	-	-	-	-	-	-
					2010	1	-	1	-	-	-	-	-	-	-	-	-	-	-	-	-
					2012	1	-	-	-	1	-	-	-	-	-	-	-	-	-	-	-
					2013	12	3	-	-	-	9	-	-	-	-	-	-	-	-	-	-
					2014	13	1	-	1	-	4	7	-	-	-	-	-	-	-	-	-
					2015	66	1	-	-	-	-	-	19	1	1	30	3	1	2	3	4
					2016	269	4	-	-	2	-	3	42	-	2	20	1	19	3	48	2
					2017	8	1	-	-	-	-	-	-	-	-	-	-	-	-	1	-
																					
8630	GTA	NS5	322	V	2006	0	-	-	-	-	-	-	-	-	-	-	-	-	-	-	-
					2010	0	-	-	-	-	-	-	-	-	-	-	-	-	-	-	-
					2012	0	-	-	-	-	-	-	-	-	-	-	-	-	-	-	-
					2013	0	-	-	-	-	-	-	-	-	-	-	-	-	-	-	-
					2014	6	-	-	-	-	-	6	-	-	-	-	-	-	-	-	-
					2015	2	1	-	-	-	-	-	1	-	-	-	-	-	-	-	-
					2016	83	-	-	-	-	-	2	1	-	-	-	-	-	-	-	-
					2017	2	-	-	-	-	-	-	-	-	-	-	-	-	-	-	-
																					
9239	TGC	NS5	525	C	2006	0	-	-	-	-	-	-	-	-	-	-	-	-	-	-	-
					2010	0	-	-	-	-	-	-	-	-	-	-	-	-	-	-	-
					2012	0	-	-	-	-	-	-	-	-	-	-	-	-	-	-	-
					2013	0	-	-	-	-	-	-	-	-	-	-	-	-	-	-	-
					2014	0	-	-	-	-	-	-	-	-	-	-	-	-	-	-	-
					2015	6	-	-	-	-	-	-	-	-	-	-	-	1	2	3	-
					2016	94	-	-	-	-	-	-	-	-	-	-	-	21	3	48	-
					2017	5	-	-	-	-	-	-	-	-	-	-	-	-	-	1	-
																					
9425	A**AA**	NS5	587	K	2006	1	1	-	-	-	-	-	-	-	-	-	-	-	-	-	-
					2010	1	-	1	-	-	-	-	-	-	-	-	-	-	-	-	-
					2012	1	-	-	-	1	-	-	-	-	-	-	-	-	-	-	-
					2013	12	3	-	-	-	9	-	-	-	-	-	-	-	-	-	-
					2014	13	1	-	1	-	4	7	-	-	-	-	-	-	-	-	-
					2015	67	1	-	-	-	-	-	20	1	1	30	3	1	2	3	4
					2016	294	4	-	-	2	-	3	54	-	2	20	1	22	3	51	2
					2017	9	1	-	-	-	-	-	-	-	-	-	-	-	-	1	-
																					
9536	AGT	NS5	624	S	2006	0	-	-	-	-	-	-	-	-	-	-	-	-	-	-	-
					2010	0	-	-	-	-	-	-	-	-	-	-	-	-	-	-	-
					2012	0	-	-	-	-	-	-	-	-	-	-	-	-	-	-	-
					2013	0	-	-	-	-	-	-	-	-	-	-	-	-	-	-	-
					2014	0	-	-	-	-	-	-	-	-	-	-	-	-	-	-	-
					2015	0	-	-	-	-	-	-	-	-	-	-	-	-	-	-	-
					2016	10	-	-	-	-	-	-	-	-	-	-	-	-	-	-	-
					2017	0	-	-	-	-	-	-	-	-	-	-	-	-	-	-	-
																					
9590	TCA	NS5	642	S	2006	1	1	-	-	-	-	-	-	-	-	-	-	-	-	-	-
					2010	1	-	1	-	-	-	-	-	-	-	-	-	-	-	-	-
					2012	0	-	-	-	-	-	-	-	-	-	-	-	-	-	-	-
					2013	11	2	-	-	-	9	-	-	-	-	-	-	-	-	-	-
					2014	13	1	-	1	-	4	7	-	-	-	-	-	-	-	-	-
					2015	64	-	-	-	-	-	-	18	1	1	30	3	1	2	3	4
					2016	275	3	-	-	-	-	3	52	-	2	20	1	20	2	42	1
					2017	9	1	-	-	-	-	-	-	-	-	-	-	-	-	1	-
																					
10088	ATT	NS5	808	I	2006	0	-	-	-	-	-	-	-	-	-	-	-	-	-	-	-
					2010	0	-	-	-	-	-	-	-	-	-	-	-	-	-	-	-
					2012	0	-	-	-	-	-	-	-	-	-	-	-	-	-	-	-
					2013	0	-	-	-	-	-	-	-	-	-	-	-	-	-	-	-
					2014	0	-	-	-	-	-	-	-	-	-	-	-	-	-	-	-
					2015	0	-	-	-	-	-	-	-	-	-	-	-	-	-	-	-
					2016	52	-	-	-	-	-	-	-	-	-	-	-	-	-	1	-
					2017	0	-	-	-	-	-	-	-	-	-	-	-	-	-	-	-
																					
10163	GCG	NS5	833	A	2006	0	-	-	-	-	-	-	-	-	-	-	-	-	-	-	-
					2010	0	-	-	-	-	-	-	-	-	-	-	-	-	-	-	-
					2012	0	-	-	-	-	-	-	-	-	-	-	-	-	-	-	-
					2013	0	-	-	-	-	-	-	-	-	-	-	-	-	-	-	-
					2014	0	-	-	-	-	-	-	-	-	-	-	-	-	-	-	-
					2015	34	-	-	-	-	-	-	-	-	-	30	3	-	-	-	-
					2016	25	-	-	-	-	-	-	-	-	-	17	1	-	-	2	-
					2017	0	-	-	-	-	-	-	-	-	-	-	-	-	-	-	-
																					
10265	AAC	NS5	867	N	2006	1	1	-	-	-	-	-	-	-	-	-	-	-	-	-	-
					2010	1	-	1	-	-	-	-	-	-	-	-	-	-	-	-	-
					2012	0	-	-	-	-	-	-	-	-	-	-	-	-	-	-	-
					2013	12	3	-	-	-	9	-	-	-	-	-	-	-	-	-	-
					2014	13	1	-	1	-	4	7	-	-	-	-	-	-	-	-	-
					2015	65	1	-	-	-	-	-	18	1	1	30	3	1	2	3	4
					2016	291	4	-	-	-	-	3	54	-	2	20	1	22	3	44	2
					2017	9	1	-	-	-	-	-	-	-	-	-	-	-	-	1	-
																					
10265	AAT	NS5	867	N	2006	0	-	-	-	-	-	-	-	-	-	-	-	-	-	-	-
					2010	0	-	-	-	-	-	-	-	-	-	-	-	-	-	-	-
					2012	1	-	-	-	1	-	-	-	-	-	-	-	-	-	-	-
					2013	0	-	-	-	-	-	-	-	-	-	-	-	-	-	-	-
					2014	0	-	-	-	-	-	-	-	-	-	-	-	-	-	-	-
					2015	2	-	-	-	-	-	-	2	-	-	-	-	-	-	-	-
					2016	2	-	-	-	2	-	-	-	-	-	-	-	-	-	-	-
					2017	0	-	-	-	-	-	-	-	-	-	-	-	-	-	-	-
																					
10298	GAG	NS5	878	E	2006	0	-	-	-	-	-	-	-	-	-	-	-	-	-	-	-
					2010	0	-	-	-	-	-	-	-	-	-	-	-	-	-	-	-
					2012	0	-	-	-	-	-	-	-	-	-	-	-	-	-	-	-
					2013	0	-	-	-	-	-	-	-	-	-	-	-	-	-	-	-
					2014	6	-	-	-	-	-	6	-	-	-	-	-	-	-	-	-
					2015	0	-	-	-	-	-	-	-	-	-	-	-	-	-	-	-
					2016	80	-	-	-	-	-	2	-	-	-	-	-	-	-	-	-
					2017	4	-	-	-	-	-	-	-	-	-	-	-	-	-	-	-
**Nt**	**Codon**	**Gene**	**Residue**	**AA**	**Year**	**N/Year**	**Venez**	**Ecuad**	**Peru**	**Nicar**	**Guade**	**DoRep**	**USA**	**Jamai**	**Marti**	**Cuba**	**Fiji**	**Samoa**	**Tonga**	**Impor**	
8006	ATG	NS5	114	M	2006	1	-	-	-	-	-	-	-	-	-	-	-	-	-	-	
					2010	1	-	-	-	-	-	-	-	-	-	-	-	-	-	-	
					2012	1	-	-	-	-	-	-	-	-	-	-	-	-	-	-	
					2013	12	-	-	-	-	-	-	-	-	-	-	-	-	-	-	
					2014	6	-	-	-	-	-	-	-	-	-	-	-	-	-	-	
					2015	1	-	-	-	-	-	-	-	-	-	-	-	-	-	-	
					2016	17	-	-	-	-	-	-	-	-	-	-	4	4	1	1	
					2017	1	-	-	-	-	-	-	-	-	-	-	-	-	-	-	
																					
8006	GTG	NS5	114	V	2006	0	-	-	-	-	-	-	-	-	-	-	-	-	-	-	
					2010	0	-	-	-	-	-	-	-	-	-	-	-	-	-	-	
					2012	0	-	-	-	-	-	-	-	-	-	-	-	-	-	-	
					2013	0	-	-	-	-	-	-	-	-	-	-	-	-	-	-	
					2014	7	-	-	-	-	-	-	-	-	-	-	-	-	-	-	
					2015	65	-	-	-	-	-	-	-	-	1	-	-	-	-	-	
					2016	256	10	3	2	20	1	27	47	1	-	-	-	-	-	2	
					2017	7	-	-	-	-	-	-	-	-	-	6	-	-	-	-	
																					
8081	CCG	NS5	139	P	2006	1	-	-	-	-	-	-	-	-	-	-	-	-	-	-	
					2010	1	-	-	-	-	-	-	-	-	-	-	-	-	-	-	
					2012	1	-	-	-	-	-	-	-	-	-	-	-	-	-	-	
					2013	12	-	-	-	-	-	-	-	-	-	-	-	-	-	-	
					2014	13	-	-	-	-	-	-	-	-	-	-	-	-	-	-	
					2015	66	-	-	-	-	-	-	-	-	1	-	-	-	-	-	
					2016	269	10	3	2	20	1	27	47	1	-	-	4	4	1	3	
					2017	8	-	-	-	-	-	-	-	-	-	6	-	-	-	-	
																					
8630	GTA	NS5	322	V	2006	0	-	-	-	-	-	-	-	-	-	-	-	-	-	-	
					2010	0	-	-	-	-	-	-	-	-	-	-	-	-	-	-	
					2012	0	-	-	-	-	-	-	-	-	-	-	-	-	-	-	
					2013	0	-	-	-	-	-	-	-	-	-	-	-	-	-	-	
					2014	6	-	-	-	-	-	-	-	-	-	-	-	-	-	-	
					2015	2	-	-	-	-	-	-	-	-	-	-	-	-	-	-	
					2016	83	-	-	-	-	1	27	46	4	-	-	-	-	-	2	
					2017	2	-	-	-	-	-	-	-	-	-	2	-	-	-	-	
																					
9239	TGC	NS5	525	C	2006	0	-	-	-	-	-	-	-	-	-	-	-	-	-	-	
					2010	0	-	-	-	-	-	-	-	-	-	-	-	-	-	-	
					2012	0	-	-	-	-	-	-	-	-	-	-	-	-	-	-	
					2013	0	-	-	-	-	-	-	-	-	-	-	-	-	-	-	
					2014	0	-	-	-	-	-	-	-	-	-	-	-	-	-	-	
					2015	6	-	-	-	-	-	-	-	-	-	-	-	-	-	-	
					2016	94	-	-	-	19	-	-	3	-	-	-	-	-	-	-	
					2017	5	-	-	-	-	-	-	-	-	-	4	-	-	-	-	
																					
9425	A**AA**	NS5	587	K	2006	1	-	-	-	-	-	-	-	-	-	-	-	-	-	-	
					2010	1	-	-	-	-	-	-	-	-	-	-	-	-	-	-	
					2012	1	-	-	-	-	-	-	-	-	-	-	-	-	-	-	
					2013	12	-	-	-	-	-	-	-	-	-	-	-	-	-	-	
					2014	13	-	-	-	-	-	-	-	-	-	-	-	-	-	-	
					2015	67	-	-	-	-	-	-	-	-	1	-	-	-	-	-	
					2016	294	10	3	2	19	1	29	49	4	1	-	4	4	1	3	
					2017	9	-	-	-	-	-	-	-	-	-	7	-	-	-	-	
																					
9536	AGT	NS5	624	S	2006	0	-	-	-	-	-	-	-	-	-	-	-	-	-	-	
					2010	0	-	-	-	-	-	-	-	-	-	-	-	-	-	-	
					2012	0	-	-	-	-	-	-	-	-	-	-	-	-	-	-	
					2013	0	-	-	-	-	-	-	-	-	-	-	-	-	-	-	
					2014	0	-	-	-	-	-	-	-	-	-	-	-	-	-	-	
					2015	0	-	-	-	-	-	-	-	-	-	-	-	-	-	-	
					2016	10	-	-	-	-	-	-	-	-	-	-	4	4	1	1	
					2017	0	-	-	-	-	-	-	-	-	-	-	-	-	-	-	
																					
9590	TCA	NS5	642	S	2006	1	-	-	-	-	-	-	-	-	-	-	-	-	-	-	
					2010	1	-	-	-	-	-	-	-	-	-	-	-	-	-	-	
					2012	0	-	-	-	-	-	-	-	-	-	-	-	-	-	-	
					2013	11	-	-	-	-	-	-	-	-	-	-	-	-	-	-	
					2014	13	-	-	-	-	-	-	-	-	-	-	-	-	-	-	
					2015	64	-	-	-	-	-	-	-	-	1	-	-	-	-	-	
					2016	275	10	3	2	18	1	28	51	3	1	-	4	4	1	3	
					2017	9	-	-	-	-	-	-	-	-	-	7	-	-	-	-	
																					
10088	ATT	NS5	808	I	2006	0	-	-	-	-	-	-	-	-	-	-	-	-	-	-	
					2010	0	-	-	-	-	-	-	-	-	-	-	-	-	-	-	
					2012	0	-	-	-	-	-	-	-	-	-	-	-	-	-	-	
					2013	0	-	-	-	-	-	-	-	-	-	-	-	-	-	-	
					2014	0	-	-	-	-	-	-	-	-	-	-	-	-	-	-	
					2015	0	-	-	-	-	-	-	-	-	-	-	-	-	-	-	
					2016	52	-	-	-	-	1	2	45	3	-	-	-	-	-	-	
					2017	0	-	-	-	-	-	-	-	-	-	-	-	-	-	-	
																					
10163	GCG	NS5	833	A	2006	0	-	-	-	-	-	-	-	-	-	-	-	-	-	-	
					2010	0	-	-	-	-	-	-	-	-	-	-	-	-	-	-	
					2012	0	-	-	-	-	-	-	-	-	-	-	-	-	-	-	
					2013	0	-	-	-	-	-	-	-	-	-	-	-	-	-	-	
					2014	0	-	-	-	-	-	-	-	-	-	-	-	-	-	-	
					2015	34	-	-	-	-	-	-	-	-	1	-	-	-	-	-	
					2016	25	-	-	2	-	-	1	1	-	1	-	-	-	-	-	
					2017	0	-	-	-	-	-	-	-	-	-	-	-	-	-	-	
																					
10265	AAC	NS5	867	N	2006	1	-	-	-	-	-	-	-	-	-	-	-	-	-	-	
					2010	1	-	-	-	-	-	-	-	-	-	-	-	-	-	-	
					2012	0	-	-	-	-	-	-	-	-	-	-	-	-	-	-	
					2013	12	-	-	-	-	-	-	-	-	-	-	-	-	-	-	
					2014	13	-	-	-	-	-	-	-	-	-	-	-	-	-	-	
					2015	65	-	-	-	-	-	-	-	-	1	-	-	-	-	-	
					2016	291	10	3	2	20	1	30	53	4	1	-	4	4	1	3	
					2017	9	-	-	-	-	-	-	-	-	-	7	-	-	-	-	
																					
10265	AAT	NS5	867	N	2006	0	-	-	-	-	-	-	-	-	-	-	-	-	-	-	
					2010	0	-	-	-	-	-	-	-	-	-	-	-	-	-	-	
					2012	1	-	-	-	-	-	-	-	-	-	-	-	-	-	-	
					2013	0	-	-	-	-	-	-	-	-	-	-	-	-	-	-	
					2014	0	-	-	-	-	-	-	-	-	-	-	-	-	-	-	
					2015	2	-	-	-	-	-	-	-	-	-	-	-	-	-	-	
					2016	2	-	-	-	-	-	-	-	-	-	-	-	-	-	-	
					2017	0	-	-	-	-	-	-	-	-	-	-	-	-	-	-	
																					
10298	GAG	NS5	878	E	2006	0	-	-	-	-	-	-	-	-	-	-	-	-	-	-	
					2010	0	-	-	-	-	-	-	-	-	-	-	-	-	-	-	
					2012	0	-	-	-	-	-	-	-	-	-	-	-	-	-	-	
					2013	0	-	-	-	-	-	-	-	-	-	-	-	-	-	-	
					2014	6	-	-	-	-	-	-	-	-	-	-	-	-	-	-	
					2015	0	-	-	-	-	-	-	-	-	-	-	-	-	-	-	
					2016	80	-	-	-	-	1	28	45	4	-	-	-	-	-	-	
					2017	4	-	-	-	-	-	-	-	-	-	4	-	-	-	-	

### Geographical patterns of emergence and persistence of pre-epidemic mutations

**Fig 1:**
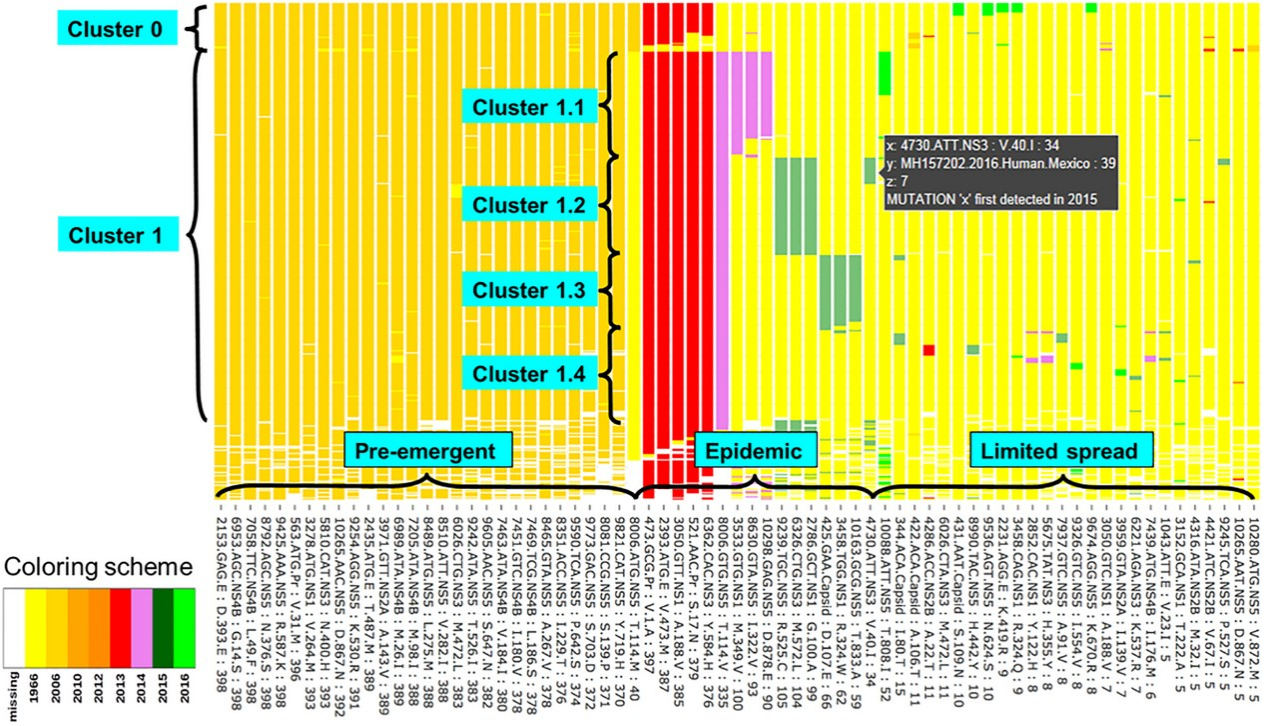
Overview of dispersal patterns of nonsynonymous mutations prior to and during the 2015–2016 ZIKV epidemic. Mutations that are detected in 5 or more genome sequences are shown on the x axis and are listed in order of number of sequences with the mutation (high to low; 398–5). The y axis represents data from 408 ZIKV genomes and due to the number of genomes the genome names are not shown in this condensed version of the figure; see Figs 2 and 3. Cells are colored according to year in which a given mutation was first detected in a ZIKV genome sequence, as shown in legend located in the lower right corner of the figure. Mutation data from Asian strain genomes are condensed to allow mutations to be grouped according to co-occurrence, prevalence, and persistence. S1 and S2 Figs include interactive versions of the data spreadsheet enabling individual cells to be queried by cursor position (as shown in brown inset adjacent to mutation queried). Readout includes: x mutation information, y sample identification, z # corresponding to color code (i.e. 0 no color, data missing), year mutation was first detected, or in cases where more than one mutation occurs at the residue “different than described” for example mutations at nts 3050, 3458, 6026, 8006, and 10265). X-axis labels include nucleotide location: codon: protein: codon mutation: number of genomes containing that mutation.

**Fig 2:**
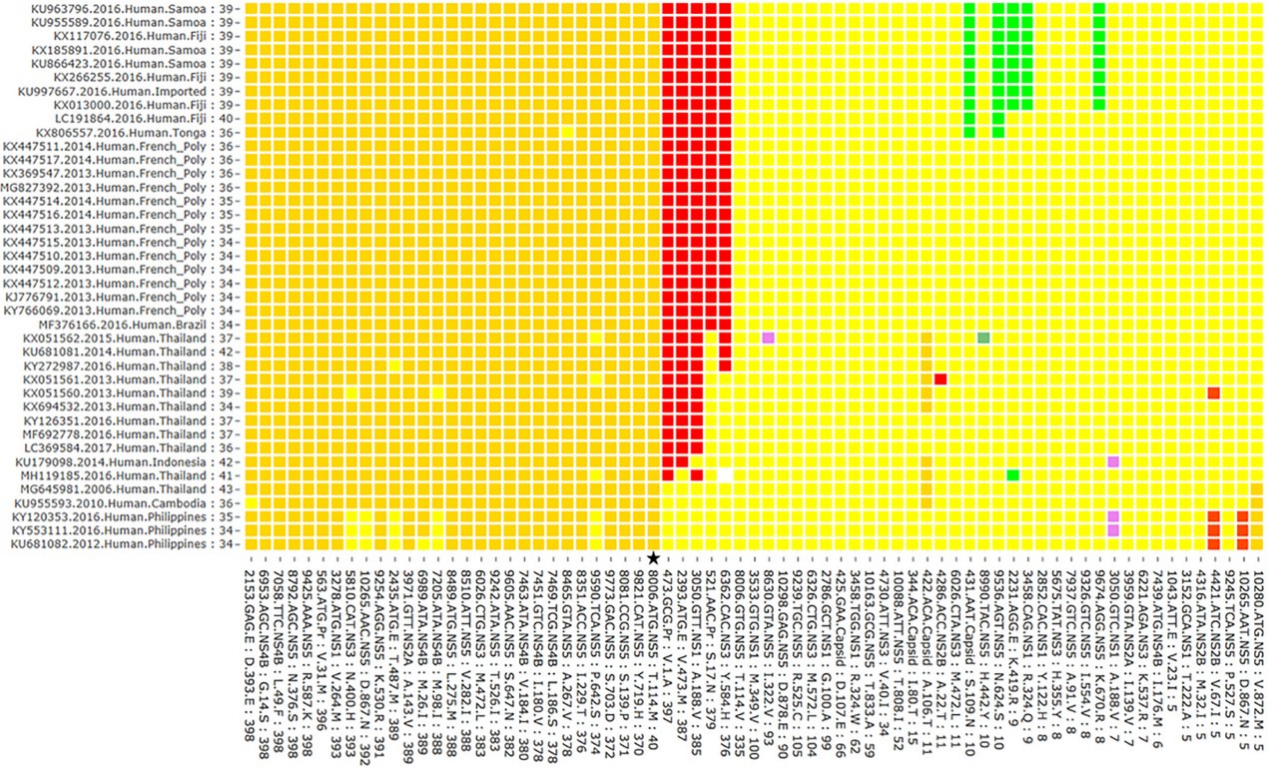
Overview of the dispersal patterns of nonsynonymous mutations within all sequences from Cluster 0. Subsection of the graph from Fig 1 showing higher resolution of mutation patterns within the Cluster 0. Cluster 0 is defined by NS5 mutation Thr114Met (marked by a star) first detected in 2006. Mutation accession numbers, year, host type, country and number of mutations detected in genomes are listed on the y axis. Genomes listed in the y axis are listed in order that best illustrates mutation clusters. Y-axis labels include: Accession number. year. species. geographic location the mutation was identified: total number of mutations identified in that genome compared to the reference genome (Malaysia 1966).

**Fig 3:**
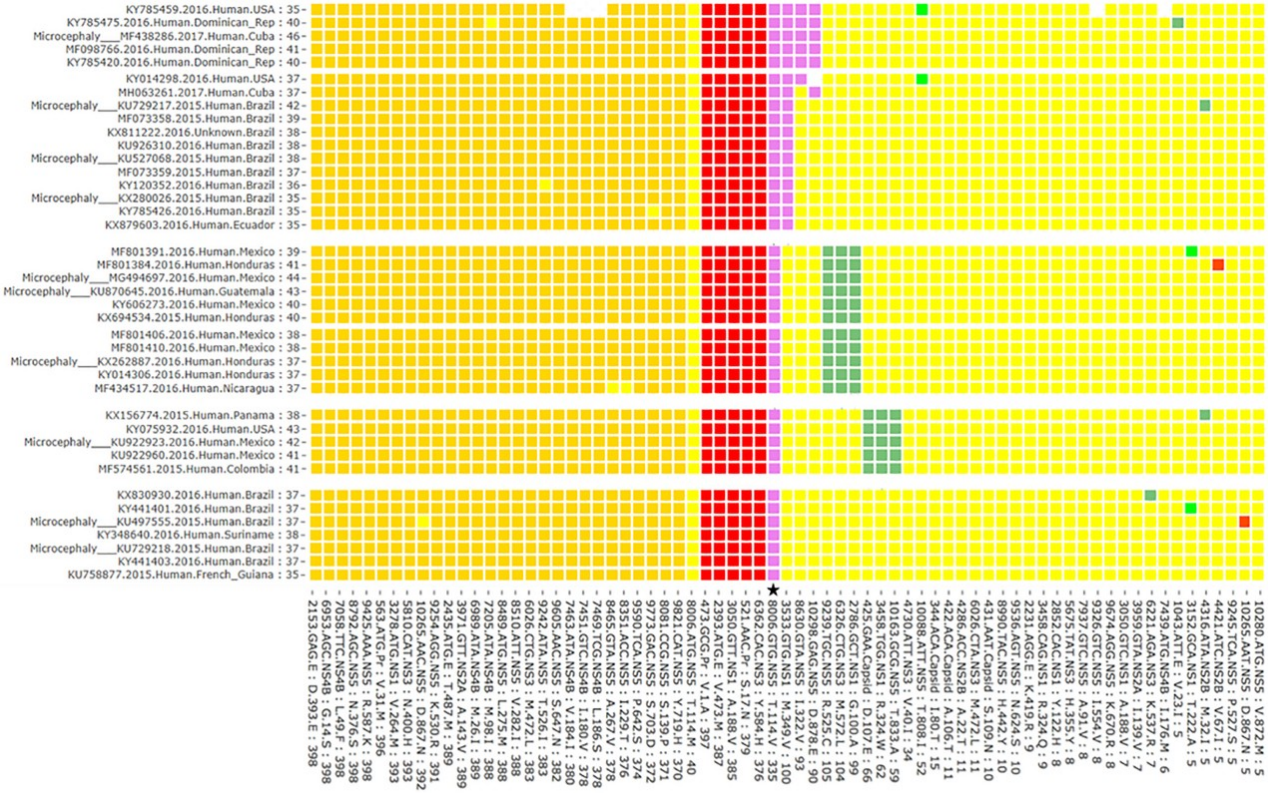
Overview of dispersal patterns of nonsynonymous mutations within sequences from Cluster 1. Subsections of the graph from Fig 1 showing higher resolution of mutation patterns. Cluster 1 is defined by NS5 mutation Thr/Met114Val (Fig 4E) first detected in 2014 (marked by a star). Fragments of clusters 1.1–1.4 with genomes listed in the y axis are selected to show the locations of sequences from microcephaly cases.

Sequence data from 408 consensus sequences and 544 mutations were organized into tables to illustrate the different temporal and geographical dispersal patterns and allow comparison of specific mutations (Tables 1–4, S1 Table). To visualize patterns of co-occurring mutations across the entire viral genome coding region, mutation data from Asian lineage genomes were condensed and sorted to allow mutations to be grouped according to co-occurrence, prevalence, and persistence (Figs 1–3). This organization of data enables observations to be made in regard to regions of the genome undergoing nonsynonymous mutation in the years leading up to the epidemic and as the epidemic progressed. For example, graphing of mutations at the amino acid level show that six mutations, NS1 Tyr122His and Met349Val, NS3 His355Tyr, and NS5 114Val (but not 114Met), Ile322Val, and Asp878Glu, were first detected in Haiti and not detected in French Polynesia in 2013 or in any of the pre-epidemic strains (S1 Table), although the number of sequenced genomes available from prior to 2013 is quite limited. This is in agreement with a phylogenetic analysis by Lednicky et al, 2016 [33], which indicated that ZIKV was circulating in Haiti in 2013, prior to emergence in Brazil. Three of these mutations, NS1 349Val, and NS5 322Val and 878Glu, were detected in relatively low numbers in Brazil but subsequently became more abundant in the Dominican Republic and USA. In addition to these genotypes being highly prevalent, as the outbreak unfolded, these mutations persisted and were all detected in one or more samples from Cuba 2017. In the case of NS5 residue 114, Table 4 illustrates the replacement of Met with Val as the epidemic progresses.

### Identification of clusters of mutations across the genome

Fig 1 illustrates the condensed version of the mutation dispersal data from S1 Table graphed according to the number of genomes with each mutation and the year in which the mutation was first observed (Fig 1, S1 and S2 Figs). This graphic representation allows clusters of mutations to be readily discerned, for example, the emergence of NS5 114Val in 2014 (and subsequent replacement of NS5 114Met which sequences form Cluster 0, see Fig 2), as well as smaller clusters of mutations that occurred from 2014 to 2016, are defined by the subsets of strains in which the mutation was detected. Because whole genome data from 408 Zika genomes are included in the graph individual data points can be defined by moving the cursor over the graph (S1 and S2 Figs). By zooming in, the graph allows individual genomes to be identified on the x axis thus allowing data points to be attributed to individual genomes from Cluster 0 (Fig 2) and Cluster 1 (Fig 3). This allows clusters of mutations to be defined by prevalence, date, and location. For example, a group on the upper right of Fig 1 were first detected in 2016 (light green bars) but did not spread extensively. By zooming in and identifying the group of sequences that had these mutations, as shown in Fig 2, one can see that these mutations were largely confined to islands in Polynesia and to Fiji. Thus, it is likely that the limited spread may have been influenced due to island location, and possibly reduced fitness associated with the mutation.

This graphic representation of large sets of sequencing data clearly shows combinations of mutations that were first detected in 2013 (Pr Val1Ala and Ser17Asn, Env Val473Met, NS1 Ala188Val, NS3 Tyr584His) and especially NS5 Thr114Val 2014 were turning point mutations and subsequently became an integral part of the epidemic genotype. This group of genomes (Cluster 1) can be divided into four subgroups defined by three sets of specific mutations first detected in 2014 and 2015. Cluster 1.1 consisting of ~100 genomes (mostly Haiti, Brazil, Dominican Republic, and USA—Florida) is characterized by one additional 2014 mutation: NS1 Met349Val. Cluster 1.2 of ~100 genomes (mostly Honduras, Mexico, and Nicaragua) is defined by three 2015 mutations: NS1 Gly100Ala, NS3 Met572Leu, and NS5 Arg525Cys. Cluster 1.3 (~60 genomes; mostly Colombia, Panama, and Venezuela) can be identified by three 2015 mutations: Capsid Asp107Glu, NS1 Arg324Trp, and NS5 Thr833Ala. And finally, the Cluster 1.4 (~100 genomes; mostly Brazil, Puerto Rico, and Venezuela) can be defined based on the absence of any of the above listed mutations (Clusters 1.1–1.3), i.e. showing the same residues at these positions as observed in the ancestral 1966 Malaysia strain. Other mutations that were detected early in the epidemic did not spread as extensively and occurred in only a handful of cases as shown by the scattered mutations on the right side of Fig 1. While those mutations that first occurred late in 2016 may have had limited transmission due to herd immunity, others may have reduced viral fitness that limited spread.

Although the clusters described in this study are similar to phylogenetic groupings from the outbreak described previously [29,34], the sheer number of samples associated with large outbreaks makes more subtle patterns of mutations difficult to visualize. This problem becomes more significant as rapid whole genome sequencing of hundreds of samples becomes the norm. Observations derived from relatively small subgroups such as these shown in this graphic representation of the large ZIKV data set may serve as starting points for hypothesis formation, particularly if there is metadata associated with sequences. For example, within Cluster 1.1 there is a group of 12 sequences that notably have two amino acids unchanged from the ancestral 1966 Malaysian strain at 2 NS5 positions, Ile322 and Asp878, and 3 of these sequences are obtained from microcephaly cases (Fig 3) suggesting this genotype be particularly virulent. Thus this combination of mutations may be interesting to target for reverse genetic characterization.

### Identification of subconsensus mutational hotspots

Genomic sequence data generally is limited to the dominant, “consensus”, sequence, however, because RNA viruses exist within a host as genetically diverse “mutant spectra” viral phenotype is affected by both consensus and variant (“subconsensus”) genotypes. In some cases variants are present at high frequency, occasionally becoming consensus; thus minor shifts in variant frequencies can sporadically affect the consensus sequence leading to an overestimation of genetic differences between isolates. This is particularly likely at regions of the genome that are known to be hypervariable. Variants can also persist at low levels within a host and serve as a reservoir of emergent genotypes, or alternatively may represent remnants of an ancestral genotype [35]. The advent of next generation sequencing technology has provided a window into the dynamics of the mutant spectra and thus may reveal unique insight into mechanisms of genotype emergence, and may provide insight into the trajectory of an outbreak.

To identify if any of the epidemic associated mutations represent “hot spots” of diversity in the genome, deep Illumina sequencing was performed on two different ZIKV strains after passage *in vivo*. A 2015 strain of ZIKV from Brazil (strain ZIKV/H.sapiens-tc/BRA/2015/Brazil_SPH2015, KU321639.1) was analyzed prior to and after passage in a pregnant macaque. Additionally, deep sequence data were obtained for a 2015 strain, PRVABC59 (accession # KU501215), from Puerto Rico before and after passage in mice dosed with Ifnar1 blocking mouse mAb (MAR1-5A3) which blocks the IFN-α/β response. PRVABC59 is a strain used in many animal infections and has been passaged multiple times in cell culture prior to the infections. However, passage in animals introduces selection pressures absent from cell culture and thus may more accurately reflect intrahost variability circulating in nature as compared to sequence data from serially passaged samples. Although necessary for productive infection, the use of mice with a compromised IFN-α/β response does introduce unnatural biases that may impact the range of variants detected in the tissues. For example, mAb MAR1-5A3 does not cross the blood-brain barrier efficiently, and this may impact viral replication in brain tissue [36].

Alternating replication of arboviruses in vertebrate and invertebrate hosts represents a distinct set of challenges for viral fitness and impacts the quasispecies composition [37,38]. In this study, the ZIKV strains were passaged in two different vertebrate hosts, unfortunately we were unable to obtain deep sequencing data from ZIKV-infected, unpassaged mosquito samples, which would have enabled analysis of variants generated during replication in the invertebrate host.

RT-PCR amplicons were generated from RNA extracted from tissue samples from a ZIKV-infected pregnant rhesus macaque [39] and from infected male mice (dosed with anti-IFNAR1 antibody MAR1-5A3, as described). These samples were deep sequenced and data were compared for the presence of variants that resulted in an amino acid change. These analyses indicated that 17 residues and 2 insertions had variants present at >1% in two or more samples tested. Six variants were present only in the macaque tissues, 7 variants were present only in the mouse samples, and 6 variants were present in tissues from both hosts (Table 5).

**Table 5:** Nonsynonymous variants detected in two or more samples during *in vivo* passage. Mutation frequencies (shown as percent) with a frequency of >1% were identified using CLC Workbench. Sample sequences were compared to the Genbank sequence for each strain to identify variant sites. Low frequency mutations were verified using overlapping read pairs (ORP) and error modeling [40]. Sites with 2 different variants present have the second variant amino acid noted next to the frequency (i.e. Thr605Ser detected for sample M4.Te at 40%). NC = no coverage at this site; LC = Low coverage (less than 300 reads). Frequencies were derived by alignment of reads to the appropriate reference sequence from GenBank. Mice are numbered 1–4. AF amniotic fluid, LN lymph node, Va vagina, Spl spleen, Te testes. Br Brain, d day post infection.

			Macaque Data					Mouse Data								
Nt	Mutation	BRZ Stock	AF d2	AF d7	LN	Va	PRV Stock	M1.Spl	M1.Te	M2.Br	M2.Spl	M2.Te	M3.Spl	M3.Te	M4.Spl	M4.Te
77	Insertion	76.9	99.4	99.3	99.7	NC										
118	Insertion		1.7	7.7		NC	7.2						1.8		1.2	
1920	Thr605Ile	7.6				NC	3.1	4.1	2.0					20.6		(Ser: 40.0)
1964	Val620Leu						83.5		82.0				49.0	47.4	20.0	20.3
2780	Trp892Gly		(Arg: 1.7)				12.6	97.7	18.5	99.5	98.9	99.0	33.3	28.3	10.8	69.9
3147	Met1014Thr						7.6	6.5	1.8				8.9		2.3	2.6
3281	Lys1059Glu						6.3	19.5	29.5				12.7	2.5	11.5	1.6
3458	Arg1118Trp		2.0			11.1										
3534	Met1143Lys		15.5	7.4		8.8										
3893	Ala1263Thr		3.6					99.0		100.0	98.5		16.8			
3894	Ala1263Val		3.6					3.2					1.2		70.4	8.3
3986	Asp1294Asn		2.5	4.2				1.2								1.6
4316	Met1404Val	(Arg: 3.7)	3.9			8.7		40.0	35.8				8.6	19.6		
4318	Met1404Ile		24.7	15.7		19.4		15.4	26.1				16.3		7.9	
5679	Ser1858Phe						55.4	2.5					11.0	40.3	2.0	14.0
6570	Thr2155Ile		9.1	12.8	8.8											
6688	Asn2194Lys	6.8	5.2	17.6			31.5									
6696	Ile2197Thr	7.4	5.4	19.3			30.7									
8156	Val2684Ile				LC		3.4	17.7	24.5				7.1	2.0	10.7	

High frequency variants were present in the stock of both viral strains, however many of the variants detected in the tissue samples were not present in the stock suggesting that these sites readily mutate during *in vivo* passage. Because the PRVABC59 GenBank sequence (accession # KU501215) used as a reference for variant detection was obtained from direct RT-PCR of the original human serum sample [41], variants detected in the stock used in this study may have arisen due to laboratory passage [42]. Alternatively, because the GenBank sequence data for KU501215 is limited to consensus data, these variants may have been in the original clinical sample as subconsensus variants but not included in the GenBank consensus data. Understanding the diversity of the mutant spectrum in clinical samples is important for understanding which mutations arise *de novo* during laboratory passage and which high frequency mutations are indeed circulating in nature.

The sequence data from the stock sample of PRVABC59 (“PRV”, Table 5) showed that while most mutations present in the stock were also detected in the mouse tissues, NS4A residues 75 and 78 (polyprotein residues 2194 and 2197, nts 6688 and 6696) had high frequency variants present at 31.5% and 30.7%, respectively that were not found in any of the mouse tissues indicating that they may be selected against during passage in mice. These NS4A variants, Asn75Lys and Ile78Thr, are also present in both amniotic fluid samples, days 2 and 7, as well as the Brazil 2015 GenBank reference sequence (accession # KU321639), thus were not selected against during passage in the macaque. NS4A is a small, hydrophobic protein, and is not well characterized with little structural data are available, so the potential impact of these mutations is unknown. Interestingly, an analogous mutation occurs with WNV with NS4A position 6687 mutating from a His to Gly after passage in mosquito cells, thus for both ZIKV and WNV there is a fluctuation at this residue between a positively charged residue and polar uncharged residue [43,44].

Two of the high frequency variants, Env Val330Lys and NS1 Trp98Gly, that were detected in the tissues of multiple mice (Table 5), were also observed as high frequency variants in a recent study by Duggal, et al., 2019 which described the passage of the same ZIKV strain, PRVABC59, in immunocompromised AG129 mice. Interestingly, the study used reverse genetics to show that the 330Leu variant had an attenuated phenotype in mice, and concluded that the Env 330Leu/NS1 98Gly genotype was enriched during passage in Vero cells and less pathogenic in mice. Thus, our data demonstrate that variant data is reproducible between laboratories and study parameters and may detect variants that impact viral pathogenicity.

Insertions and deletions are often detected as variants, sometimes in multiple samples and studies. For example, an insertion at nt 117–118 causing a single nucleotide frameshift in the capsid protein at residue 7 is observed in a number of samples. This mutation was detected in samples from the macaque (amniotic fluid, 1.7% and 7.7%), 2 of the mouse spleen samples (1.8% and 1.2%) as well as in the sequence data from the French Polynesia strain (2.2%) in the SRA (Bioproject PRJNA316976).

### Variants that become consensus sequences

To determine if mutations associated with the 2015–2016 epidemic also exist as variants, variant sequence data from laboratory stock derived from human cases and from our *in vivo* studies were compared to consensus data deposited in GenBank. Additionally, when available, deep sequence data from pre-outbreak strains (i.e. Univ. Wisconsin Bioproject PRJNA316976) were queried for the presence of variants that became consensus during the epidemic. Subconsensus mutations were detected throughout the genome, and many appeared in multiple samples and in some cases become consensus. For example, at nt 860, a variant in M protein residue, Phe37Leu, is detected at approximately 1.6% in one macaque amniotic fluid sample, and is consensus in a single sequence from an epidemic sample from 2016, but does not persist as consensus in other epidemic samples. Data from Univ. of Wisconsin Bioproject PRJNA413108 shows that a Puerto Rico ZIKV laboratory strain has 37Leu at 17% and the variant was also detected in stock of Paraiba strain at 9.4% [45], thus indicating that this mutation may be present as a variant in a variety of sample types but seldom reaches consensus.

**Fig 4:**
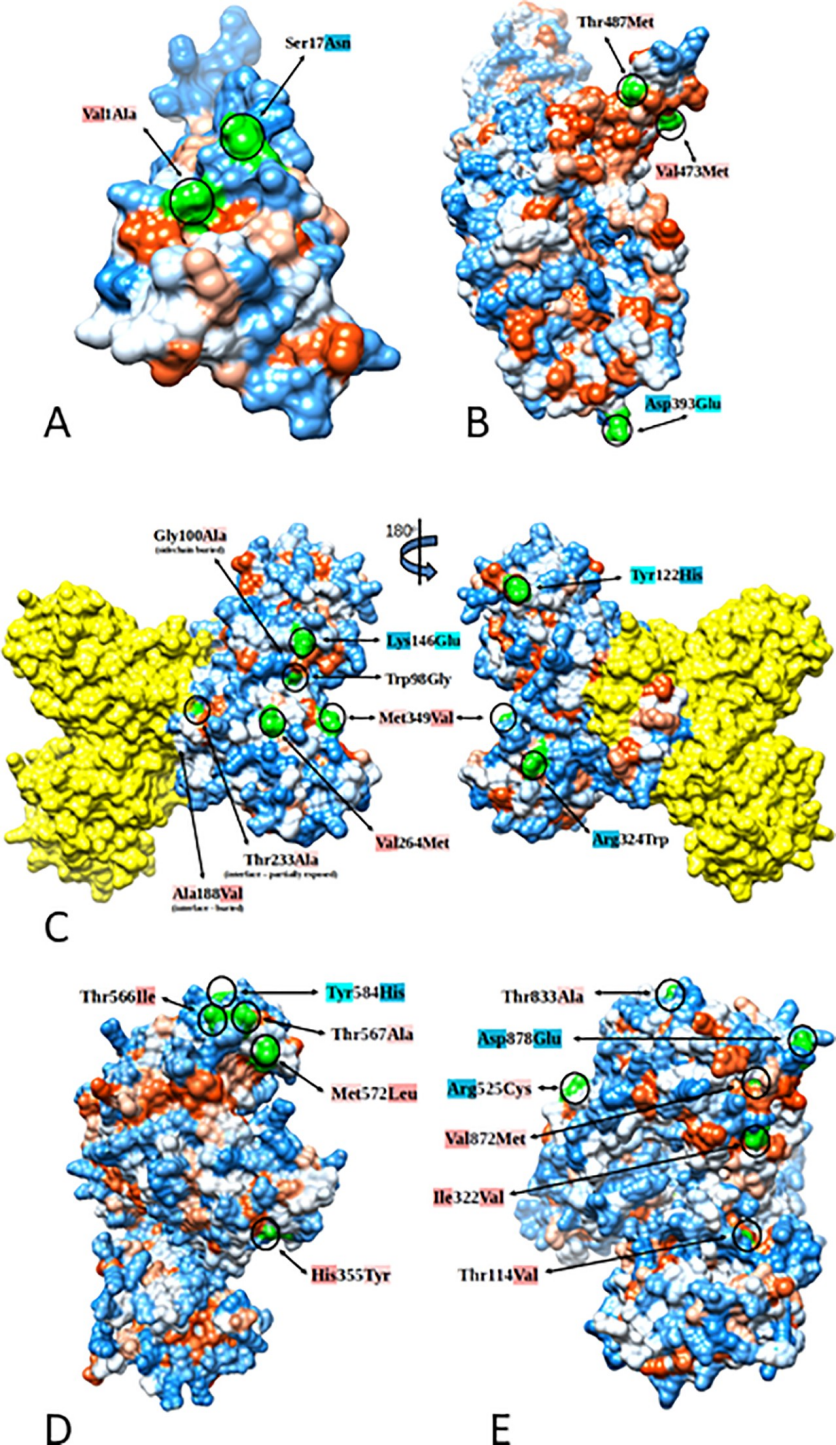
Epidemic-associated mutations tend to occur in exposed regions of Zika proteins. Protein “hydrophobicity surface” representation of Zika proteins Pr, E, NS1, NS3, and NS5 using Kyte-Doolittle scale [47] and the coloring scheme from blue (hydrophilic) to red (hydrophobic), with white color representing neutral residues. For a given mutation, a difference in hydrophobicity score between initial residue and the mutation is indicated by a change in the color intensity used as text background. For example, light red for lower and dark red for higher hydrophobicity, and light blue for lower and dark blue for higher hydrophilicity. Exposed regions of listed mutations are circled and colored in green. Plots were constructed using Chimera software. (A) Model of the Pr protein with epidemic mutations first detected in 2013: Val1Ala, Ser17Asn. (B) E protein with high abundance epidemic mutation Val473Met (observed in 387 sequences; see Table 1) first detected in 2013, and two mutations, Asp393Glu and Thr487Met, first observed in pre-emergent samples from Thailand in 2006, Cambodia in 2010, Philippines in 2012 and French Polynesia in 2013. (C) Homodimer conformation of the NS1 protein (chain B colored in yellow). Mutations Met349Val, Gly100Ala, and Arg324Trp are from different subclusters: 1.1, 1.2, and 1.3, respectively (Fig 1, Table 2). Mutation Tyr122His first detected in Haiti 2014 and Brazil 2015, and a group of three exposed mutations and in close vicinity to each other with significant amino acid property change observed: Trp98Gly, Lys146Glu, and Val264Met. (D) NS3 protein showing 4 microcephaly associated mutations Thr566Ala, Thr567Ile, Met572Leu and Tyr584His, with predicted location in the interface with NS5, and His355Tyr mutation first detected in Haiti 2014, Brazil 2015, and not detected in French Polynesia. (E) NS5 protein showing critical outbreak mutation 114Val (forms Cluster 1; Fig 1 and Table 4), Ile322Val mutation with predicted location in the interface with NS3, and epidemic mutations Arg525Cys, Thr833Ala, and Asp878Glu from different subclusters: 1.2, 1.3, and 1.4, respectively (Fig 1 and Table 4).

Some residues had two or more variants present, indicating the propensity of the protein to tolerate variation at this site. For example, at NS1 residue 98 (polyprotein residue 892; Fig 4C) a macaque amniotic fluid sample had a Trp98Arg mutation detected at low level. Interestingly, a low frequency 98Arg mutation has also been detected in a human clinical sample [46]. However, in mouse samples, a Trp98Gly variant was detected at very high frequency (10.8%-99.5%) in all nine mouse tissue samples tested and became consensus sequence in five of these samples. One consensus sequence from Puerto Rico 2015 also had a Gly mutation present at this residue (S2 Table). Protein structural modeling indicates that this residue is exposed, thus these mutations may occur in response to interaction with the host immune system. Interestingly, neighboring mutation Gly100Ala becomes consensus during the outbreak and increases in prevalence from 8 to 24% from 2015–16 (Fig 4).

### Genetic variation at residues associated with the ZIKV epidemic genotype

Many of the mutations that characterize the ZIKV 2015–2016 epidemic genotype show variation at the subconsensus level. In some cases, an amino acid that was previously consensus in pre-outbreak samples was detected at subconsensus levels in epidemic strains. For example, nt 473, Pr residue 1, changes from a Val to an Ala in 2013, and Ala persists during the outbreak as consensus. Interestingly one amniotic sample from the ZIKV-infected macaque has a Val present at 1% indicating that ancestral variants may be present at low frequency. Additionally, Val is present at 3.5% in data from a macaque infected with the French Polynesia strain from Bioproject PRJNA316976.

Similarly, at NS1 residue 264 (polyprotein residue1058), there is a change from Val to Met in Thailand 2006 and Cambodia 2010, and Met is present as consensus in samples from pre-epidemic and epidemic cases, whereas the Malaysia strain has Val (S2 Table). However, Val is still present as a variant in samples prior to and during the epidemic. Val is detected at 4.2% in Illumina data from French Polynesia stock virus (Bioproject PRJNA316976) and maintained during passage in two macaques at 4.2 and 9.9%.

NS1 mutation Arg324Trp (polyprotein residue 1118) was observed in a single sample from Brazil but spread extensively to other countries during the outbreak, in particular Colombia as shown in Cluster 1.3 and Table 2. A different amino acid, 324Gln, was detected in several 2016 cases, however, only 324Trp mutation was detected as a variant in macaque amniotic and vaginal tissues at 2.0% and 11.1% (Table 5), respectively. Interestingly SRA data from Bioproject PRJNA316976 show the 324Trp mutation present in an infected macaque at 6.5%, but not in the French Polynesia stock used to initiate the infection. Protein structure analysis shows residue 324 is exposed on the surface of NS1 (Fig 4C) and a mutation from a charged residue, Arg, to a nonpolar amino acid, Trp, may impact protein-protein interactions.

The NS1 mutation 349Val (polyprotein residue1143) emerged in 2014 and spread extensively during the epidemic (Table 2), however this residue varies in consensus and variant sequences of pre-epidemic and epidemic strains. For example, strains from Africa, Malaysia, Cambodia, and French Polynesia have Met349 as consensus, whereas 2014 strains from Haiti have either Met or Val, as do outbreak strains from Brazil. Interestingly, Val at residue 349 has been associated with at least 4 microcephaly cases (3 Brazil 2015 and 1 Cuba 2017) [4,32,45]. Deep sequence data show that this residue also varies at the subconsensus level, but not necessarily reflecting the pre-outbreak amino acid, Val. For example, analysis of the Illumina data from the 2013 French Polynesia strain (Univ. of Wisconsin Bioproject PRJNA316976) show a Thr variant present at 7.3% in stock virus and up to 10.4% after passage in macaques. Our data from macaque passage of the Brazil 2015 strain (accession # KU321639) shows Met349Lys at 7.4% to 15.5% in amniotic samples and at 8.8% in vagina samples (Table 5). The mutational profile of the Paraiba strain shows a Val to Ala mutation at a frequency of 4.2% [45]. Structural analysis [48] shows that residue 349 is exposed on the protein surface (Table 6, Fig 4C), thus it is possible that this residue is variable in response to host immune selection pressures. Reverse genetics studies using *in vivo* models are required to determine the effect of the various mutations at this site on viral phenotype.

**Table 6:** Structural analysis of mutations associated with epidemic trajectory and microcephaly. The percentage of mutations occurring in greater than 10% of ZIKV genomes (Asian Lineage) between 2010–2017, with at least 25 cases occurring in 2016, is shown in middle column “%(2006–17)”. Mutations that are predicted by protein structural models to occur in exposed regions of the protein are marked as a “1” in the “Exposed” column, and mutations predicted to occur in buried regions are marked as “0”. Cells with the 5 mutations that occurred in 2013 (as shown in Fig 1) are red, and cells representing the 2014 NS5 Thr114Val mutation are light purple. Cells with Cluster 1.1 mutation NS1 Met349Val are pink, and Cluster 1.2 mutations and Cluster 1.3 mutations are in blue and green cells, respectively. Mutations that are found in the whole genome sequences of all known microcephaly cases are in bold italicized text in the “Epi Mut” column and bold black text in this column marks mutations observed in at least 1 microcephaly case. Black underlined text marks mutations that are not observed in genome sequences from any microcephaly cases.

Nt	Residue	Gene	Gene Residue	Epi Mut	Prev AA	Exposed	%(2006–17)	#(2006–17)	#2006	#2010	#2012	#2013	#2014	#2015	#2016	#2017
425	107	Capsid	107	**E**	D	1	16.3	66	-	-	-	-	-	33	33	-
473	123	Pr	1	***A***	V	1	98.2	397	-	-	-	12	13	67	295	10
521	139	Pr	17	***N***	S	1	95.4	379	-	-	-	9	11	66	284	9
563	153	Pr	31	***M***	V	1	100.0	396	1	1	1	12	13	67	291	10
2153	683	E	393	***E***	D	1	99.7	398	1	-	1	12	13	67	294	10
2393	763	E	473	***M***	V	1	98.4	387	-	-	-	12	13	67	286	9
2435	777	E	487	***M***	T	1	98.9	389	1	1	-	12	13	67	286	9
2786	894	NS1	100	**A**	G	1	25.0	99	-	-	-	-	-	6	88	5
3050	982	NS1	188	***V***	A	1	97.4	385	-	-	-	12	12	67	285	9
3278	1058	NS1	264	***M***	V	1	100.0	393	1	1	1	12	13	67	288	10
3458	1118	NS1	324	**W**	R	1	15.6	62	-	-	-	-	-	35	27	-
3533	1143	NS1	349	**V**	M	1	25.3	100	-	-	-	-	6	5	85	4
3971	1289	NS2A	143	***V***	A	0	99.7	389	1	1	1	12	13	66	285	10
5810	1902	NS3	400	***H***	N	1	98.7	393	1	1	-	11	13	67	290	10
6026	1974	NS3	472	***L***	M	1	97.2	383	1	1	1	12	13	67	278	10
6326	2074	NS3	572	**L**	M	1	26.3	104	-	-	-	-	-	6	94	4
6362	2086	NS3	584	***H***	Y	1	96.1	376	-	-	-	9	12	66	280	9
6953	2283	NS4B	14	***S***	G	1	100.0	398	1	1	1	12	13	67	293	10
6989	2295	NS4B	26	***I***	M	1	97.0	389	1	1	-	12	12	63	290	10
7058	2318	NS4B	49	***F***	L	1	99.5	398	1	1	1	12	13	67	293	10
7205	2367	NS4B	98	***I***	M	0	97.9	388	1	1	-	11	13	67	285	10
7451	2449	NS4B	180	***V***	I	0	100.0	378	1	1	1	12	13	67	274	9
7463	2453	NS4B	184	***I***	V	0	100.0	380	1	1	1	12	13	67	275	10
7469	2455	NS4B	186	***S***	L	0	99.7	378	1	1	1	12	13	67	273	10
8006	2634	NS5	114	**M**	T	1	10.6	40	1	1	1	12	6	1	17	1
8006	2634	NS5	114	***V***	T	1	89.1	335	-	-	-	-	7	65	256	7
8081	2659	NS5	139	***P***	S	1	99.4	371	1	1	1	12	13	66	269	8
8351	2749	NS5	229	***T***	I	0	98.1	376	1	1	1	12	13	67	272	9
8465	2787	NS5	267	***V***	A	1	96.9	378	1	1	1	12	13	67	273	10
8489	2795	NS5	275	***M***	L	1	100.0	388	1	1	1	12	13	67	283	10
8510	2802	NS5	282	***I***	V	0	100.0	388	1	1	1	12	13	67	283	10
8630	2842	NS5	322	**V**	I	1	24.2	93	-	-	-	-	6	2	83	2
8792	2896	NS5	376	***S***	N	1	99.7	398	1	1	1	12	13	67	295	8
9239	3045	NS5	525	**C**	R	1	27.0	105	-	-	-	-	-	6	94	5
9242	3046	NS5	526	***I***	T	1	98.7	383	1	1	1	12	13	66	280	9
9254	3050	NS5	530	***R***	K	1	99.7	391	1	1	1	12	13	67	287	9
9425	3107	NS5	587	***K***	R	1	100.0	398	1	1	1	12	13	67	294	9
9590	3162	NS5	642	***S***	P	1	97.4	374	1	1	-	11	13	64	275	9
9605	3167	NS5	647	***N***	S	1	100.0	382	1	1	1	12	13	66	279	9
9773	3223	NS5	703	***D***	S	1	99.7	372	1	1	1	12	13	67	267	10
9821	3239	NS5	719	***H***	Y	1	100.0	370	1	1	1	12	13	67	266	9
10088	3328	NS5	808	**I**	T	1	13.3	52	-	-	-	-	-	-	52	-
10163	3353	NS5	833	**A**	T	1	15.0	59	-	-	-	-	-	34	25	-
10265	3387	NS5	867	***N***	D	1	98.7	392	1	1	-	12	13	65	291	9
10298	3398	NS5	878	**E**	D	1	22.7	90	-	-	-	-	6	-	80	4

NS2B residue 32 (polyprotein residue 1404), shows significant variation at the consensus and subconsensus level. Mutations Met32Val and Met32Ile are detected in both the macaque and mouse tissue, at up to 40% and 26%, respectively (Table 5). Interestingly, Illumina deep sequence data available from a 2013 French Polynesia strain also showed a Met32Ile mutation detected at 5% (Bioproject PRJNA316976). These mutations did become consensus in several epidemic samples, in particular 4 sequences from 2015 and 3 from 2016 show an Ile as consensus, and one 2016 sample had Val as consensus (S2 Table). Unfortunately, this residue falls within a region of the NS2B proteins that is not amendable to reliable protein structural models.

### Structural analysis of mutations associated with epidemic trajectory and microcephaly cases

Computational system, GeneSV [49], was used to facilitate assessment of regions of sequence/structure variability in ZIKV genomic sequences. Such assessments may be very valuable especially for RNA viruses, which are characterized by high mutation rates and presence in highly diverse populations. The GeneSV system combines information from a large variety of sources and processes information from all 3 types of data: genomic sequences, corresponding protein sequences as well as homologous protein sequences, and protein structures taken (if available) from Protein Data Bank (PDB) or constructed using AS2TS homology-based protein structure modeling system [50]. Generated results aid functional annotation of genomes, predict potential nucleic and amino acid mutations not observed in current databases, and may be useful for prioritization of mutations for further study using reverse genetics.

In the present study a combination of sequence and structure based analyzes was used to identify common features of mutations associated with epidemic spread. Protein structural analysis of mutations detected in Asian linage ZIKV strains collected between 2010 and 2017 was performed to determine if the mutations that were highly transmitted in 2016 were more likely to be surface exposed (solvent accessible) or buried within the protein. Notably, most (38/45) of the mutations associated with the epidemic transmission occurred in exposed residues (Table 6, Fig 4). For example, all 4 of the mutations preceding the 2013 outbreak: Ser17Asn of the Pr protein (Fig 4A), Val473Met of the E protein (Fig 4B), Val264Met of the NS1 protein (Fig 4C), and Tyr584His of the NS3 protein (Fig 4D) (polyprotein residues Ser139Asn, Val763Met, Val1058Met, and Tyr2086His, respectively) are predicted to occur in exposed regions.

Protein structure analysis of highly transmitted mutations (occurred in 20 or more cases in 2016) showed that these mutations were more likely to occur on the surface of the protein as compared to those mutations that were not seen during 2016 or detected in fewer than 5 cases (p = 0.096, Mann Whitney two tailed t test). Interestingly, several of the mutations including 4 associated with microcephaly cases (Thr566Ala (Guatemala, 2016), Thr567Ile (Cuba, 2017), Met572Leu (Mexico, 2016), Tyr584His (Brazil, 2015)) occur in the exposed region of NS3 (residues 565–584) that interfaces with NS5 (residues 317–338) and may impact replication [51] (Fig 4D).

Combining prevalence and persistence data with information derived from protein structural modeling may provide unique context in regard which mutations may play key roles in the epidemic phenotype. For example, early in this study, protein structure analysis of mutations in both dimer and hexamer states for NS1 predicted that mutations which were associated with a microcephaly case mostly occur on exposed regions of the protein. Exceptions include Gly100Ala which occurs in a sidechain buried within the protein, and two mutations Ala188Val and Thr233Ala that are in the interface between subunits (Fig 4C). Mutation 188Val (buried within the interface) was of particular interest as it was first detected in Thailand and French Polynesia in 2013 and was maintained throughout the epidemic, thus is a mutation that defines the epidemic genotype. Recently 188Val has been shown to impact viral phenotype, increasing the replication rate in mosquitoes and decreasing host interferon response [22,52]. Mutation Val264Met is an example of highly abundant and surface exposed mutation that is also predicted to be antigenic. It was first detected in Cambodia in 2010 and thus was circulating well before the onset of the epidemic so its relevance is less obvious.

## Conclusion

The objective of this study was to examine genotype emergence at the microscale (molecular) and macroscale (temporal and spatial dispersion of mutations) to enable trends in mutations and outbreak trajectory to be assessed. At the molecular scale, this study used computational analysis of online and laboratory-generated sequence data to detect patterns of ZIKV evolution at the inter- and intra-host level. Genetic data from the recent ZIKV epidemic was examined for patterns in mutations at the protein, nucleotide, and quasispecies (variant) level. It is now acknowledged that RNA viruses evolve as a diverse intra-host population (“mutant spectrum” or “quasispecies”), and it is the diversity at the population level that determines the outcome of the infection [53,54]. The advent of next generation sequencing (NGS) enables viral populations to be defined at increasing depths and thus allows unprecedented examination of the evolutionary process. However, generation and analysis of NGS data can be complex due to the massive output of relatively short sequencing reads of the Illumina platform, which is currently the only NGS technology with error rates low enough to allow detection of low frequency variants in intra-host viral populations. Importantly, the inherently stochastic process of RNA genome replication and the resulting genetic drift produces many mutations that are lethal, as well as many which individually have no phenotypic consequence. This obscures the identification of mutations that do have functional consequences, and the short reads of Illumina sequencing make linking mutations difficult. The challenge with the current technology is to find ways to see signal and patterns through the noise. Encouragingly, as one examines deep sequence data from various sources, trends do appear, and some sites do consistently undergo mutation and the consensus and subconsensus level. As scientists increasingly deposit NGS sequence data online, it becomes possible to compare results across studies and verify patterns of mutations and hypotheses.

In this study, publicly available NGS data generated over the course of the epidemic combined with *in vivo* laboratory data allowed comparison of trends in mutations. Our *in vivo* data from ZIKV passage in a pregnant macaque and in mice confirmed the presence of mutations at subconsensus levels in ZIKV strains ZikaSPH2015 and PRVABC59, respectively, both of which were isolated from patients during outbreaks. Interestingly, some of the mutations occurred in different laboratory host species from multiple studies and were also detected as changes in the consensus sequence in a subset of epidemic samples. Similarly, amino acids that were previously consensus in pre-outbreak samples were detected as low frequency variants in epidemic strains.

Classification of measures of amino acid properties such as charge, antigenicity and conservation may also be useful for detecting trends in the data. Nonsynonymous mutations that defined the epidemic genotype were further characterized at the protein structure level. Comparative analyses of constructed protein structure models of different ZIKV variants were used to identify mutations likely to modify protein structure conformation. For example, our structural analyses indicate that mutations that were persistent and prevalent in the outbreak more often occurred in surface-exposed rather than buried regions of the protein. Interestingly, four mutations associated with microcephaly cases were shown by protein structure analysis to occur in an exposed region of NS3 that interfaces with NS5 and may impact replication. Thus, protein structural models can be used to identify mutations of potential importance such as those that occur in or adjacent to regions known to influence protein functions and/or viral phenotype.

To gauge trajectory of an outbreak it is important to look at viral mutation dynamics in the spatio-temporal context (“macroscale”) as well as in a molecular context such as the potential impact on protein functionality and viral phenotype. In this study, a process was developed for organization of large sets of sequence data, thus enabling patterns emergence and spread of mutations associated with the outbreak to be readily visualized. At the macroscale level, consensus data was organized into large and interactive databases to allow the spread of individual mutations and combinations of mutations to be visualized and assessed for temporal and geographical patterns. This type of analysis highlights the dynamics of amino acid changes within the circulating genotype, as was the case with NS5 position 114 and mutations from Thr to Met and Val between 2014 to 2016 (Table 4, Fig 1). Although many of the early mutations highlighted in this analysis were first described by Faria et al. (2016) and others, the present analysis includes sequence data from samples collected as the outbreak progressed, thus provides a comprehensive picture of clusters of mutations that persisted into later stages of the outbreak as well as mutations that were only detected in a handful of samples. Additionally, this graphical representation allows gaps in whole genome sequence data to be easily recognized.

In summary, the recent ZIKV outbreak was analyzed using next generation sequencing for detection of emerging mutations, structural models to identify mutations likely to impact viral phenotype, and organization of large datasets to detect trends in mutation spread. Together these multiscale analyses provide a mechanism estimating the trajectory of an epidemic. Use of multiscale modeling for prioritizing mutations according to geographical spread and potential for phenotypic impact can aid the formation of hypotheses which can then be tested using reverse genetics.

## Materials and methods

### Graphical spatio-temporal and genetic representation of mutations

A set of all available ZIKV complete genome sequences was collected from GenBank and ViPR databases. The sequences of Asian lineage strains were split into three groups: unassociated with the recent emergence of ZIKV (Asian strain Malaysia 1966 (accession # KX377336)), “pre-emergent strains”, and Asian genomes associated with the epidemic. The genomic sequence PRVABC-59 (accession # KU501215) was selected as a reference sequence in all comparisons to provide uniform genome coordinates (position numbering) for nucleotide sites, polyprotein residue, protein residue information, and data from protein structural analysis. Asian strain Malaysia 1966 (accession # KX377336) was used as a distant progenitor for comparison and was used as a reference to identify all mutations for further evaluation. Protein sequence-based and structure-based position conservation analyses were performed on all protein sequences collected from evaluated genome sequences (see section below, “Protein structure modeling”). Selected results from calculated characteristics of “epidemic related” mutations are provided in S3 Table. This data was used as an input for plots presented in Figs 1–3 that were created using an “in-house” code written in R with a “plotly” library to facilitate interactive analysis [55].

### Virus propagation

ZIKV strain PRVABC59 (Human/2015/Puerto Rico) was obtained from BEI Resources (NR50240 ZIKV). The complete genomic sequence of PRVABC59 has been determined (GenBank: KU501215), and the complete coding sequence of NR50240 has also been determined (GenBank: KX087101). The PRVABC59 virus stock was passaged a total of 5 times in Vero cells prior to sequencing according to the GenBank description of KX087101. The ZikaSPH2012015 (GenBank: KU321639.1) was obtained from Mike Busch at Blood Systems Research Institute and was originally isolated from a transfusion recipient in Brazil in 2015. This strain was passaged 3 times in Vero cells prior to use in this study.

### *In vivo* studies

#### Mouse infection

These studies were carried out in strict accordance with the recommendations in the *Guide for the Care and Use of Laboratory Animals* and the National Institute of Health. All efforts were made to minimize suffering of animals. All animals were housed in ABSL2 conditions in an AAALAC-accredited facility, and the protocol was approved by the LLNL Institutional Animal Care and Use Committee (IACUC), which includes ethics in evaluation of protocols. Inbred, barrier-housed, specific pathogen-free C57Bl/6J mice were obtained from (Stock 000664), and acclimated for one week prior to experiments. Six-week old, male mice were treated with an Ifnar1blocking mouse mAb (MAR15A3, Cat# I401, low endotoxin-formula, Leinco Technologies) [56,57] by intraperitoneal injection (2.5 mg/mouse), 24 hours prior to ZIKV infection. ZIKV infection (PRVABC59 strain) was injected subcutaneously/intra-dermally into the hock region of the animal [58,59] at 1 x 10^3 plaque forming units (PFU). At 7 days post-infection, tissues were collected immediately after euthanasia (brain, testis, spleen) and stored in RNA-Later (Qiagen, Valencia, CA) according to manufacturer’s instructions until RNA isolation and RNA analysis. RNA was extracted from mouse tissues using the RNeasy Mini Kit (Qiagen) according to manufacturer’s instructions, after being homogenized in lysis buffer using 1.5 and 3.0 mm Zirconium Lysis BeadBug beads and a BeadBug 3 homogenizer (Benchmark Scientific, Edison, NJ).

#### Macaque infection

In a study performed at the California National Primate Research Center, and approved by the University of California Davis Institutional Animal Care and Use Committee, a pregnant rhesus macaque (*Macaca mulatta*) was inoculated intramniotically and intravenously in the first trimester at gestation day 41 (term is 165 days). The inoculum was 5.0 log_10_ PFU of ZikaSPH2015 in 1 ml of RPMI1640 medium and was injected intravenously (IV) to simulate direct IV mosquito feeding in the saphenous vein of the animal under aseptic conditions. The same dose was also administered just after the IV inoculation via ultrasound-guided intra-amniotic inoculation under aseptic conditions. Clinical data and viral RNA tropism are described elsewhere [39]. ZIKV RNA was extracted from snap frozen tissue samples using the MagMax viral RNA kit (Thermo Fisher Scientific, Waltham, MA, USA), as described previously [60] and were frozen at 80°C until used in this study.

### Viral RNA quantitation by quantitative real time polymerase chain reaction (qRT-PCR)

#### Primer design

Primer sets were assembled that spanned a majority of the genome yielding RTPCR products of 1 or 2 kb, and which overlapped by approximately 100 nt. Primer sets included primers described in literature [61] and online (https://zika.labkey.com/project/OConnor/begin.view) previously shown to amplify epidemic ZIKV strains. Two distinct multiplexed primer sets were used to enable each region of the genome to be covered by at two different primer sets. Because we mixed subsets of primers and primers from different publications into multiplexed reactions occasionally new primers needed to be designed. The additional primers were designed to perform as two highly multiplexed reactions as described in Gardner *et al*., 2014 [62].

#### RT-PCR

Reverse transcription was performed using random hexamers and the Superscript III RT reverse transcriptase kit (Thermo Fisher Scientific). Viral cDNA templates were amplified using Q5^®^ Hot Start High Fidelity 2X Master Mix (New England BioLabs, Ipswich, MA, USA), following manufacturer’s instructions. PCR conditions consisted of 98°C for 30 s, followed by 35 cycles of 98°C for 10 s, 60°C for 20 s, and 72°C for 1 min. The final cycle was 72°C for 2 min. PCR products were prepared for sequencing using the QIAquick PCR Purification kit (Qiagen, Aarhus, Denmark).

### Viral genome sequencing

#### Illumina sequencing

RT-PCR products that were generated with each primer set (1 kb set and 2 kb set) were processed separately to enable primer output to be assessed. The 12 kb amplicons were sonicated using an M220 ultrasonicator (Covaris, Woburn, MA, USA) and final sheared sizes ranged from 144 to 186 bp with an average size of 164 bp. Libraries were prepared using the TruSeq DNA PCR-Free LT Library Preparation Kit (Illumina, San Diego, CA, USA) following standard manufacturer’s protocols with the exception of the bead-based library size selection steps in which 150 bp sized libraries were selected instead of the standard kit sizes. Indexed samples were pooled and sequenced on the Illumina MiSeq system with the MiSeq V2 sequencing kit utilizing 2 X 150 bp paired-end reads. Sequences were de-multiplexed and trimmed using the Illumina bcl2fastq conversion software. Error rate due to PCR and sequencing was controlled for using a plasmid control of known sequence and overlapping read pair analysis as described previously [40].

Illumina MiSeq sequences were imported as fastq files into CLC Genomics Workbench version 11 (Qiagen). Reads were mapped to the reference sequence of the ZIKV strained used for infection. Only nucleotide positions be covered by > 100 reads, with a base quality score >Q30 were used for variant calls.

### Protein structure modeling

In an attempt to characterize possible phenotypic changes that could be induced by specific mutations (i.e. if they are located within functional sites, binding sites or interfaces between interacting molecules), a large number of structural models of ZIKV proteins (with and without mutations) were constructed in their monomeric, oligomeric states and formed complexes. For those of ZIKV proteins for which a set of “good” homology (at least 50% of sequence identity, and 50% coverage) structural templates from Protein Data Bank (PDB) were detected, structural models were constructed using the homology based structural modeling system AS2TS ([49]; http://proteinmodel.org/). A list of primary templates from PDB used for modeling ZIKV proteins: Capsid, Pr, M-E (complex), NS1, NS3, and NS5 included respectively: 5z0r, 6idi, 6co8, 5k6k, 5y4z, and 5u0b. For proteins NS2A, NS2B, NS4A, and NS4B for which low coverage or no homology to protein structures from current PDB was detected, a number of additional “threading-based” and “de novo” models were constructed using I-TASSER and QUARK servers ([63]; https://zhanglab.ccmb.med.umich.edu/I-TASSER/). These additional constructed models were used to assess confidence in predictions of spatial location of mutations identified in proteins with low or no homology to reliable structural templates.

Created structural models were analyzed to assess the possibility of conformational changes implied by the identified epidemic mutations, and to estimate the level of possible sequence variability in detected structurally conserved regions. Structure alignments were calculated using the program LGA (Local Global Alignment) [48] and evaluation of structural similarities between ZIKV proteins and related structures from PDB was performed by StralSV sequence/structure variability evaluation system [64]. StralSV identifies structurally similar protein structure fragments in the PDB for any given structural motif, evaluates calculated structure-based alignments between the query motif and the fragments, and quantifies observed sequence variability at each residue position. This helps detection of other proteins (sometimes from more distant organisms) in which a similar structural motif with a given substitution was observed and characterized. For proteins with specific mutations additional structural models were also constructed and processed.

The collected genomic sequences and results from sequence and structure-based analyses were processed using the GeneSV system to identify unique (outbreak related) mutations and their context within ZIKV genome (i.e. sequence/structure variability and conservation regions, surface exposure). Examples of the GeneSV output is provided in S1 and S2 Tables.

## Supporting information

S1 Fig Interactive overview of dispersal patterns of nonsynonymous mutations prior to and during the 2015–2016 ZIKV epidemic. Individual cells to be queried by cursor position (as shown in brown inset adjacent to mutation queried). Readout includes: x mutation information, y sample identification, z # corresponding to color code (i.e. 0 no color, data missing), year mutation was first detected, or in cases where more than one mutation occurs at the residue “different than described” for example mutations at nts 3050, 3458, 6026, 8006, and 10265). X-axis labels include nucleotide location: codon: protein: codon mutation: number of genomes containing that mutation.
(HTML)

S2 Fig Condensed, interactive version of the mutation dispersal data prior to and during the 2015–2016 ZIKV epidemic. Individual cells to be queried by cursor position (as shown in brown inset adjacent to mutation queried). Readout includes: x mutation information, y sample identification, z # corresponding to color code (i.e. 0 no color, data missing), year mutation was first detected, or in cases where more than one mutation occurs at the residue “different than described” for example mutations at nts 3050, 3458, 6026, 8006, and 10265). X-axis labels include nucleotide location: codon: protein: codon mutation: number of genomes containing that mutation.
(HTML)

S1 Table Dispersal patterns of nonsynonymous mutations according to country and year. The number of whole genome sequences present in detected in 3 or more sequences in GenBank and ViPR databases for each epidemic-associated mutation were tabulated to allow easy visualization of similarities in time, location, and number of cases between mutations. The first column identifies each mutation according to nt site: codon: protein: residue #: amino acid. Cells are colored according to number of genome sequences with the mutation (red, yellow, green: high, medium, low). Country names are abbreviated using the first 5 letters of the name or common abbreviation. AA- amino acid; Impor–Imported case.
(XLSX)

S2 Table Compilation and comparison of nonsynonymous mutations according to protein structural context, and prevalence prior to and during the 2015–2016 ZIKV epidemic. Genome coordinates such as nucleotide site are numbered according to reference sequence PRVABC-59, accession # KU501215. Amino acid mutations are listed by polyprotein residue (“PolyRes”), and gene (“Residue”) information, and data from protein structural analysis. Mutation frequencies are listed according to year, frequency, and percent of genomes with the mutation. Column “2006–2017” shows cumulative data from 2006–2017. Other abbreviations: SS–residue is in a secondary structure element, C—coil, E—strand, H—helix; AC–residue is in an exposed region (0 = no; 1 = yes); EP–residue is in a region predicted to be an epitope (0 = no; 1 = yes). Ref AA- Amino acid present in 1966 Malaysian strain; Epi AA- Amino acid mutation present in pre-emergent (Thailand, Cambodia, Philippines) or epidemic strains. Standard single-letter amino acid and nucleotide codes are used where applicable.
(XLSX)

S3 Table List of nonsynonymous mutations according to Asian lineage strains of the 2006–2017 ZIKV. Amino acid epidemic related mutations (“Epi_AA”) are listed by gene name (“Gene”) and a residue number (“Residue”) for each ZIKV strain within which they are observed. Genome coordinates such as a nucleotide site (“Nt”) and a residue number within a gene are numbered according to reference sequence PRVABC-59, accession # KU501215. A number of genomes (“2006_17_Number_of_strains_with_Epi_AA_mutation”) where a particular mutation is observed is also listed.
(XLSX)
